# The Alpha Keto Amide Moiety as a Privileged Motif in Medicinal Chemistry: Current Insights and Emerging Opportunities

**DOI:** 10.1021/acs.jmedchem.0c01808

**Published:** 2021-03-25

**Authors:** Marco Robello, Elisabetta Barresi, Emma Baglini, Silvia Salerno, Sabrina Taliani, Federico Da Settimo

**Affiliations:** † Synthetic Bioactive Molecules Section, LBC, NIDDK, NIH, 8 Center Drive, Room 404, Bethesda, Maryland 20892, United States; ‡ Department of Pharmacy, University of Pisa, Via Bonanno 6, 56126 Pisa, Italy

## Abstract

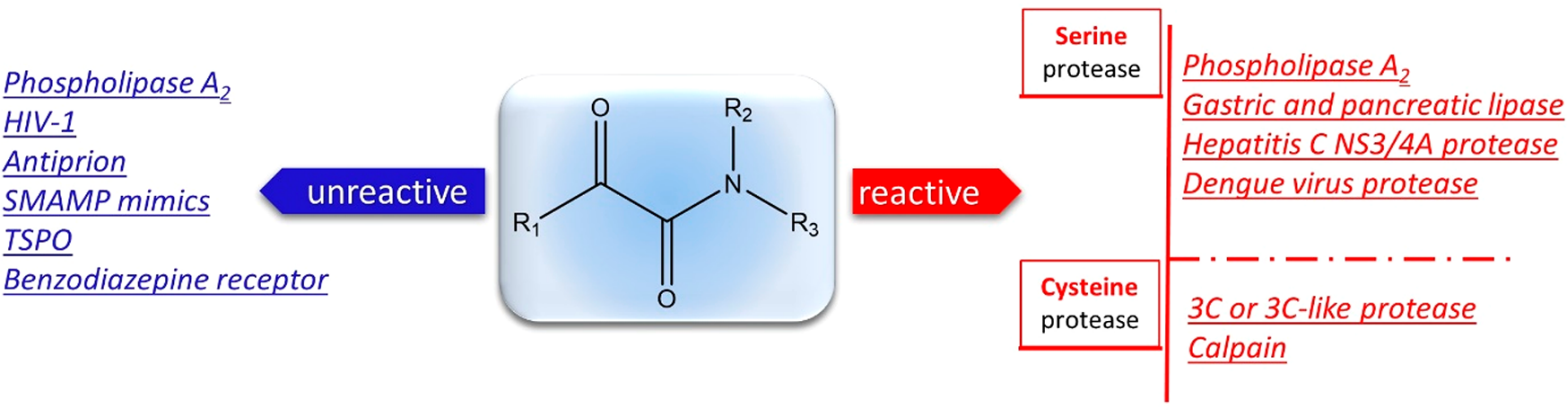

Over the years, researchers in drug discovery have taken advantage of the use of privileged structures to design innovative hit/lead molecules. The α-ketoamide motif is found in many natural products, and it has been widely exploited by medicinal chemists to develop compounds tailored to a vast range of biological targets, thus presenting clinical potential for a plethora of pathological conditions. The purpose of this perspective is to provide insights into the versatility of this chemical moiety as a privileged structure in drug discovery. After a brief analysis of its physical–chemical features and synthetic procedures to obtain it, α-ketoamide-based classes of compounds are reported according to the application of this motif as either a nonreactive or reactive moiety. The goal is to highlight those aspects that may be useful to understanding the perspectives of employing the α-ketoamide moiety in the rational design of compounds able to interact with a specific target.

## 1 Introduction

In 1988, Evans and colleagues introduced the concept of “privileged structure” to define structures able to provide useful ligands for different target proteins (receptors, enzymes, and so on) to medicinal chemistry. Moreover, intelligent modifications of these structures often represented a good strategy for the development of molecules with different efficacy profiles, such as receptor agonists and antagonists.^1^

**Figure 1:**
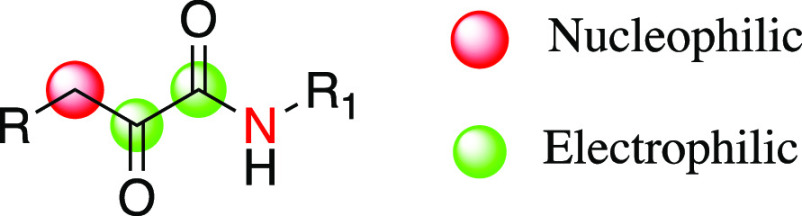
Potential reaction sites in α-ketoamides.

The α-ketoamide is a peculiarly reactive ambident proelectrophile and pronucleophile moiety, displaying two possible nucleophilic reaction sites together with two electrophilic centers (Figure 1), whose reactivity can be augmented through the selection of specific activation modes.^2^

### 1.1 Molecular Geometry

**Figure 2:**
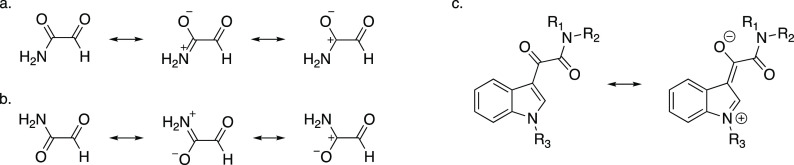
(a) Resonance forms of s-*cis*-2-oxoacetamide. (b) Resonance forms of s-*trans*-2-oxoacetamide. (c) Resonance forms of indolglyoxylamides.

The α-ketoamide preferred geometry provides that the nitrogen atom and two carbonyl groups are all on the same plane, with the two oxygen atoms in a *trans* disposition, mainly because of the mutual repulsion by the oxygen lone pairs that occurs in *cis* conformation. The two conformations present different calculated carbon–carbon bond length values (1.52-1.54 Å in the s-*trans* conformers and 1.54−1.55 Å in s-*cis* forms), which are never overcome by bond length in twisted intermediate geometries, suggesting no resonance contribution to the interaction between the two carbonyl groups, albeit the geometrical alignment may indicate so.^3^ Compared to the experimentally determined length of an amide bond in classical gaseous amides, the amide carbon–nitrogen bond in s-*trans* α-ketoamides is slightly shorter. Shortness in the C–N bond and the lack of conjugation between the two carbonyl groups suggest that α-ketoamides are comparable to amides substituted by an electron-withdrawing carbonyl group.^3^ Analyzing the interactions between the amide and keto groups led to some interesting observations. For example, if the two carbonyl groups adopt the s-*cis* conformation, a stretching of the amide bond arises, and the nitrogen becomes more negatively charged compared to the s-*trans* conformer, resulting from the diminished contribution of the resonance form where the nitrogen atom is formally double bonded to carbon (Figure 2a). On the other hand, the same resonance form becomes important in s-*trans* α-ketoamides since it reduces the electrostatic repulsion between negatively charged nitrogen and the distal oxygen (Figure 2b).^3^ It should be noted that in the specific case of indolglyoxylamides (Figure 2c), the resonance form directly affects the reactivity of the moiety, as discussed later in the manuscript.

**Figure 3:**
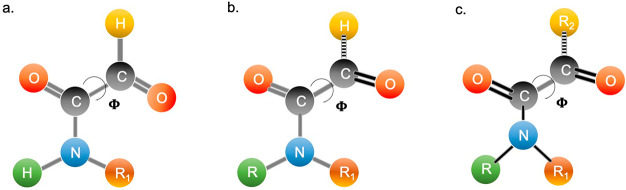
(a) Preferred conformation of the α-ketoamide with the nitrogen on the same plane of two carbonyls in trans position (dihedral angle phi = 180°). Unsubstituted (R_1_ = H) and monosubstituted nitrogen is associated with a trans conformation of the amide bond, especially if R_1_ is a small aliphatic chain. (b) If R_1_ is a bulky substituent or both R and R_1_ are different from hydrogen, together with the presence of a hydrogen atom on the distal carbonyl, the dihedral angle becomes twisted (phi = 140–150°), and the moiety loses its planarity. The nitrogen center is not affected. (c) If R_1_ is a bulky substituent or both R and R_1_ are different from hydrogen, while R_2_ is not a hydrogen atom, the dihedral angle phi is more twisted (100–140°) with consequent pyramidalization of the nitrogen center.

Computational studies elucidated that the α-ketoamide moiety prefers to adopt a planar conformation, with the nitrogen center on the same plane of the two carbonyls disposed in *trans* conformation (Figure 3a). Monosubstitution of the nitrogen center is related to a preferred *trans* geometry for the amide bond, especially if the substituent is a small aliphatic chain (Figure 3a). Since the planarity of a dicarbonyl unit is influenced by the bulkiness of the two substituents, it has been demonstrated that bulky monosubstitutions, as well as a tertiary nitrogen center, together with the presence of a hydrogen atom on the distal carbonyl, modify slightly the OC–CO dihedral angle (140–150° vs 180°), affecting the planarity of the moiety but not the planarity of the nitrogen (Figure 3b). In the case of substitution also on the distal carbonyl, a more pronounced modification of the OC–CO dihedral angle (100–140°) is present, with consequent pyramidalization of the nitrogen center and an even less planar α-ketoamide moiety (Figure 3c). A OC–CO twisted dihedral angle is also responsible for diminished strength of the C–N bond.^3^

### 1.2 Synthesis and Nomenclature

**Figure 4:**
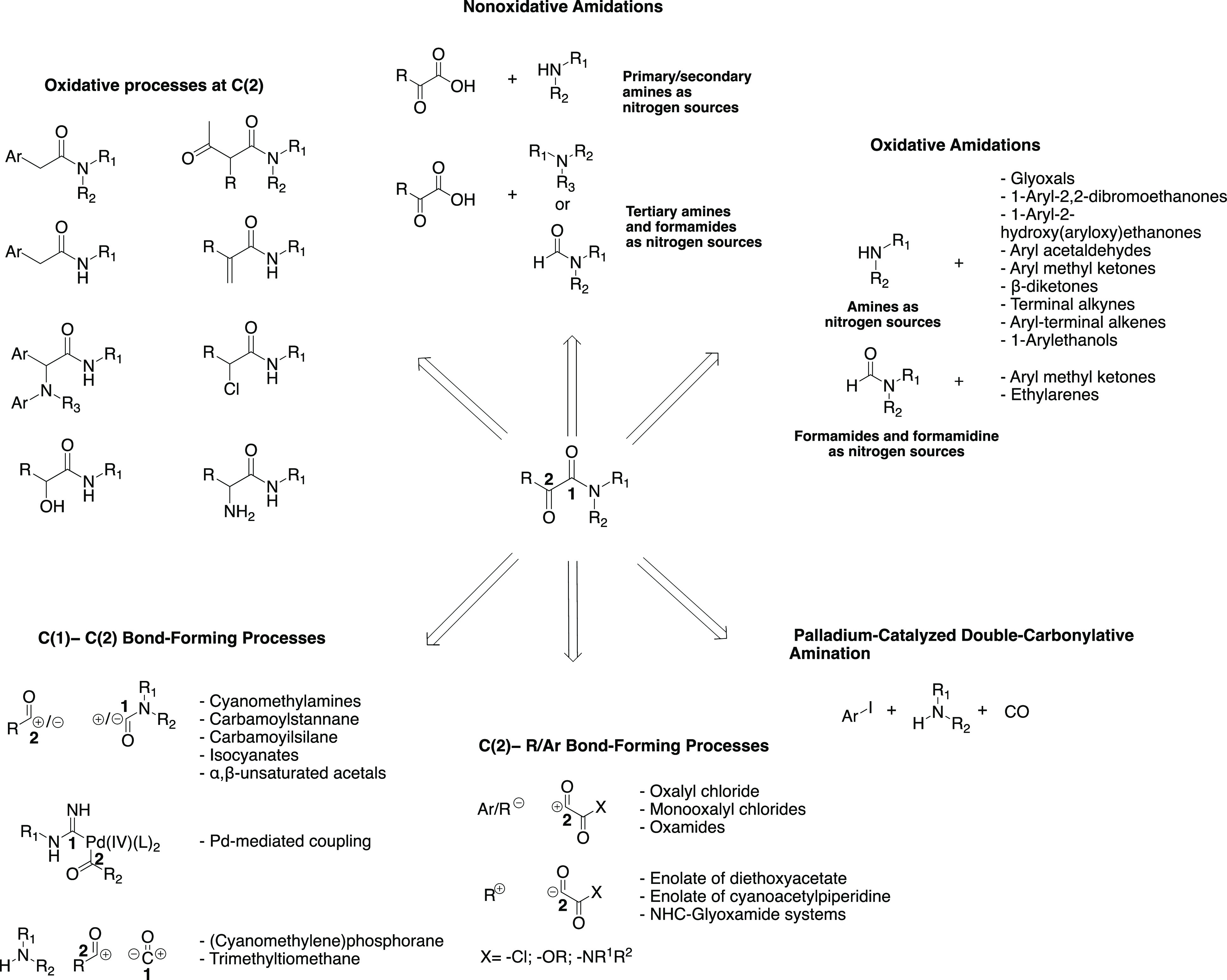
General retrosynthetic approaches for the formation of the α-ketoamide moiety.^2^

α-Ketoamides have been investigated through the decades by organic chemists for their peculiar reactivity and chemical versatility.^4−8^ These investigations have led to the development of an extraordinary variety of synthetic methods to obtain derivatives featuring this moiety. Since it is not the aim of this work to describe all of the progress made in this field, it is recommended to refer to some of the very comprehensive publications in which the most recent synthetic approaches are covered. These approaches range from C(2)-oxidation of amide starting compounds and amidation, through methodologies centered on the C(1)–C(2) σ-bond and C(2)–R/Ar bond-forming processes, to the palladium catalyzed double-carbonylative amination reactions (Figure 4).^2^

This structural motif has been reported in the literature with different nomenclatures as α-ketoamide, 2-ketoamide, 2-oxoamide, glyoxamide, and glyoxylamide. For the sake of clarity, all the variations that are presented herein are in accordance with the original articles.

### 1.3 Reactivity and Metabolic Stability

Compared to other dicarbonyl derivatives, such α-ketoacids and α-ketoesters, α-ketoamides have been shown to possess better pharmacokinetic properties. They showed improved membrane permeance compared to α-ketoacids and enhanced stability toward plasma esterases than α-ketoesters.^9,10^ α-Ketoamides are also reported to be more resistant against proteolytic cleavage.^3^ In a series of calpain inhibitors developed by different research groups, α-ketoamides have been proposed to possess superior chemical and metabolic stability compared to the aldehyde derivatives, which can give undesired reactions because of their high reactivity with the nucleophilic amino or thiol groups of various biological substances.^10−13^ This has been suggested, for example, by Zeng et al., who investigated enterovirus 71 3C protease inhibitors and discovered a series of α-ketoamide derivatives with comparable potency to inhibitors carrying an aldehyde warhead but lacking the toxicity of this highly reactive moiety.^14^

In the specific case of indolglyoxylamides, which are deeply discussed in the present manuscript, chemical stability and reduced reactivity seemed to be attributable to the character of vinylogous amide or enamide of the carbonyl directly attached to the indole ring, as exemplified by the development of fostemsavir, an HIV-1 attachment inhibitor (vide infra).^15^

When in the presence of a chiral center adjacent to the keto-carbonyl, an aspect that should be taken into consideration is the possibility of epimerization/racemization due to the electrophilicity of the carbonyl itself. Fast epimerization/racemization at physiological pH and in the presence of buffered solutions has been reported^9,16^ as well as during the synthesis^17,18^ and purification^9^ of α-ketoamide derivatives. This susceptibility could raise concerns about derivatives requiring an absolute configuration to express their biological activity, and it should be taken into consideration during the design and biological evaluation of such compounds. Another aspect about chemical reactivity of this moiety, which should be considered because it could affect synthesis, purification, and biological activity, is the possibility to form hemiacetals by reaction with water or alcohols. In aqueous medium, the keto-carbonyl can exist in the *gem*-diol hydrate form, whose stability and equilibrium with the keto form have been reported as influenced by pH and grade of substitution of the nitrogen of α-ketoamide moiety itself.^16^

Like other drugs containing a carbonyl function, the cytosolic stability of α-ketoamides can be limited by carbonyl-reducing enzymes. Such enzymes, which include medium-chain (MDR) and short-chain (SDR) dehydrogenase/reductase, aldo-keto reductase (AKR), and quinone reductase (QR), are ubiquitous in humans, and their presence has been established in several tissues such as liver, lung, brain, heart, kidney, and blood. This wide distribution is because carbonyl reduction constitutes a decisive step in Phase I metabolism: aldehyde, ketone, or quinone moieties of carbonyl-containing drugs are converted to alcohols to facilitate the elimination by Phase II conjugation or direct excretion.^19,20^

### 1.4 Natural α-Ketoamides and Analogues

**Figure 5:**
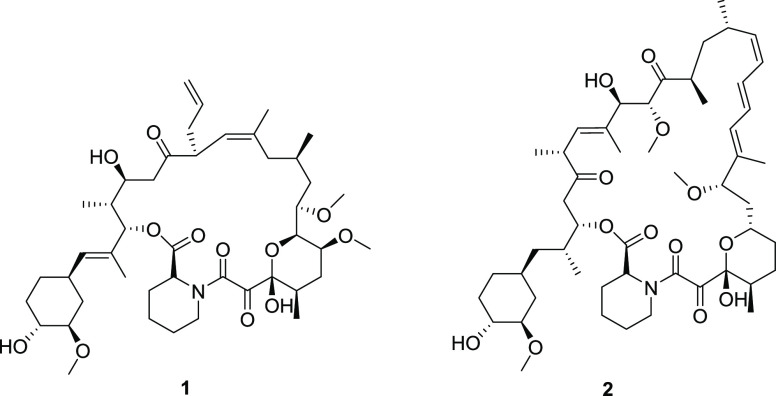
Structure of macrolides FK-506 **1** (tacrolimus)^21^ and rapamycin **2** (sirolimus).^22^

The α-ketoamide motif is a key component of several natural products, approved drugs, and drug candidates with significant biological activities. Its importance dates to the discovery of two natural products showing immunosuppressant activity: FK-506 **1**, a 23-membered macrolide lactone isolated from *Streptomyces tsukubaensis*,^21^ and rapamycin **2**, a macrolide isolated from *Streptomyces hygroscopicus* (Figure 5).^22^

These two compounds are bifunctional in nature and possess two distinct binding domains. These domains are an immunophilin binding region, which binds to FKBP12 (FK506 binding protein), and an effector domain, which mediates the interaction of the drug–immunophilin complex with the secondary protein target.^23^ Inhibition of calcineurin and RAFT (rapamycin and FKBP12 target) by these complexes is at the basis of the mechanism for the immunosuppression activity of **1** and **2**, respectively.

Crystal structure analysis of **1** and **2** complexed with FKBP12^24^ evidenced the presence of two key hydrogen bond interactions: one between the backbone NH of Ile-56 and the pipecolinic ester carbonyl and one between the amide carbonyl and Tyr-82. Additionally, a small hydrophobic, electropositive cavity is formed by Tyr-26, Phe-36, and Phe-99.

FKBP12 belongs to a wide family of chaperones of the immunophilin class that are involved in several cellular functions.^25^ It facilitates the correct folding of different proteins by catalyzing the interconversion of *cis* and *trans* amide bond rotamers in proline-containing substrates (PPIases or rotamase activity).^24^ Additionally, the immunophillins have been associated with recovery from neuronal injury^26^ and exploited as targets for the promotion of neurite outgrowth and neurotrophic and neuroprotective effects.^27^

**Figure 6:**
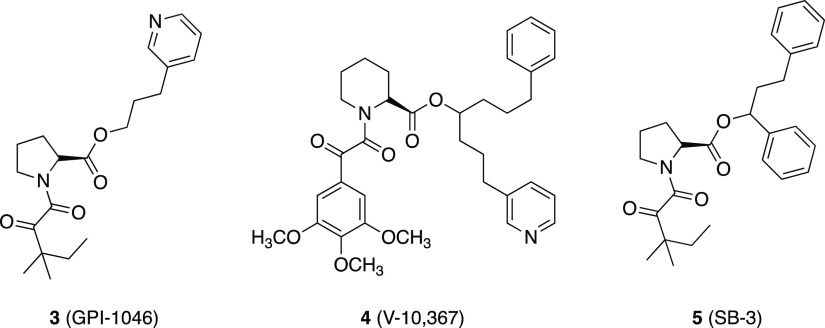
Structures of FKBP12 ligands **3**–**5**.^28−31^

Since **1** possesses neutrophic properties in vitro and in vivo, which are not caused by the effector region responsible for the immunosuppression, several compounds, such as GPI-1046 **3**,^28,29^ V-10,367 **4**,^30^ and SB-3 **5**(31) (Figure 6), were reported mimicking the only FKBP12-binding portion of **1** without the structural requirements for calcineurin inhibition. These compounds are characterized by the lack of immunosuppressant activity but are extraordinarily potent neurotrophic agents in vitro and promote neuroregeneration in vivo.^32^

X-ray and NMR structural data of these compounds complexed with FKBP12 pointed out the crucial role of α-ketoamide as nonelectrophilic moiety; indeed, hydrogen bonding interactions exist between the amide carbonyl oxygen and the Tyr82 hydroxyl group and between the ketone carbonyl oxygen and the Tyr26 hydroxyl group.^33^

**Figure 7:**
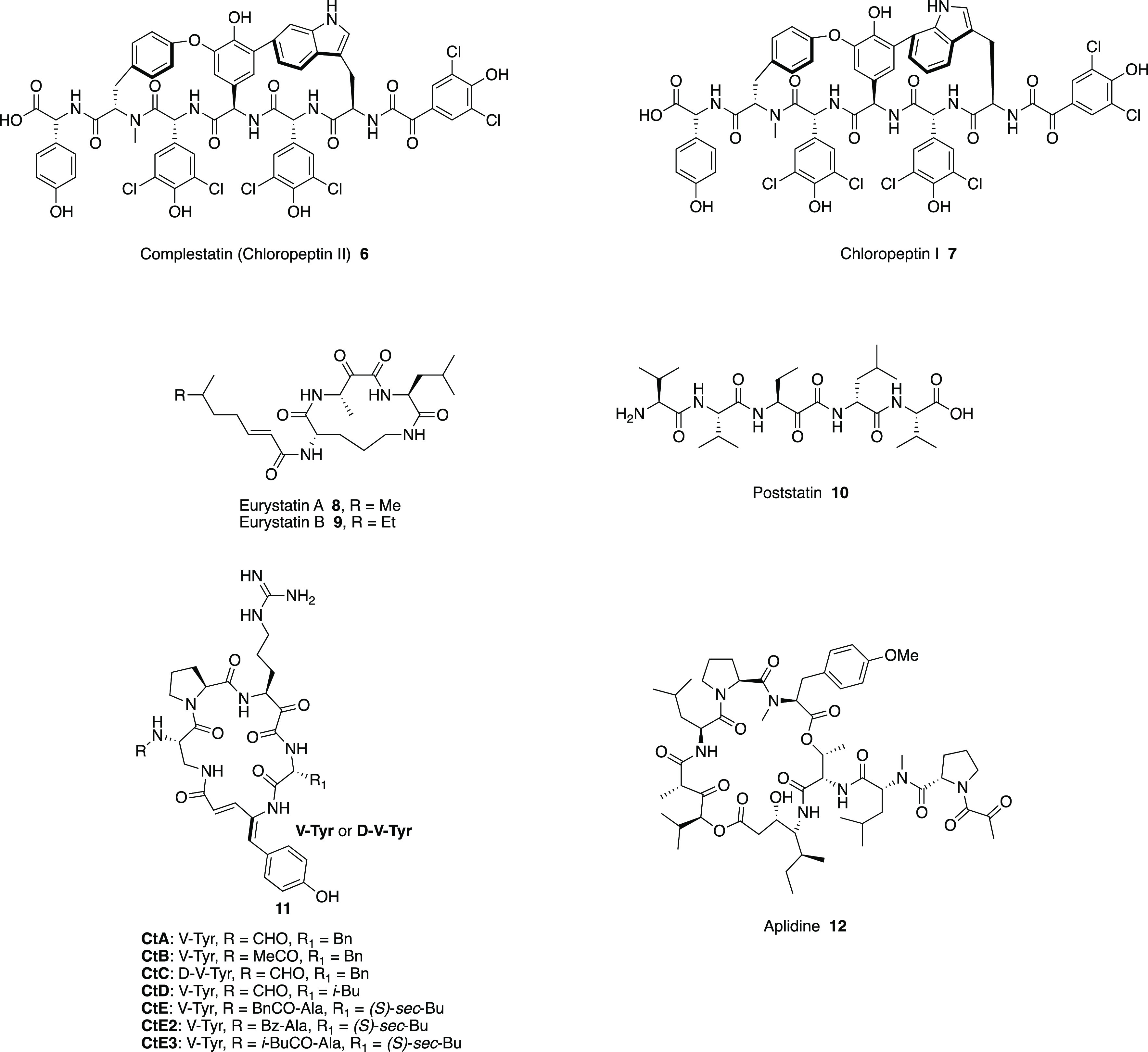
Structures of chloropeptin II (**6**)^34^ and I (**7**),^35^ eurystatins A and B (**8** and **9**, respectively),^36^ poststatin (**10**),^37^ cyclotheonamides A-E3 (**11**),^38,39^ and aplidine (**12**).^2^

**Figure 8:**
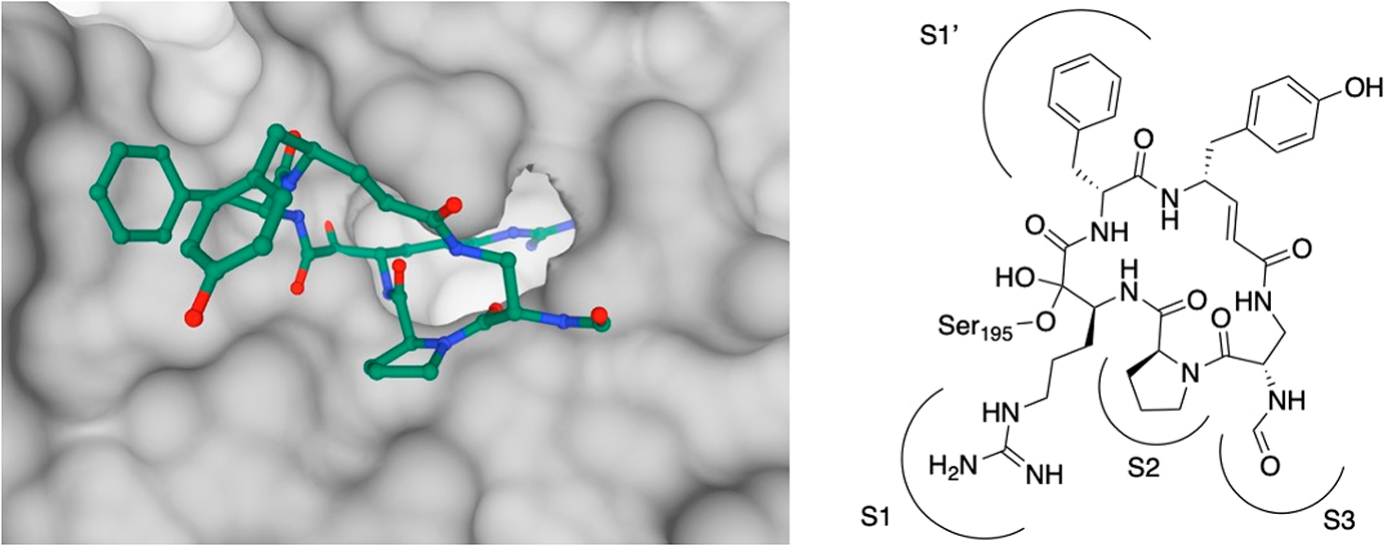
X-ray structure of trypsin in complex with cyclotheonamide A (PDB ID: 1TYN).^42,43^ (Left) View along the active site cleft into the direction of the S1 binding pocket. Trypsin is shown in gray Connolly surface representation, cyclotheonamide A in stick representation (C: green, O: red, N: blue; hydrogen atoms are omitted for clarity). (Right) Schematic representation of the binding mode; hemiketal formation with the γ-oxygen atom of Ser195 is indicated.

In addition to the macrolides **1** and **2**, other α-ketoamides from natural sources include complestatin (chloropeptin II, **6**, Figure 7), first isolated from the mycelium of *Streptomyces lavendulae* SANK 60477,^34^ and its isomer chloropeptin I (**7**, Figure 7), obtained from *Streptomyces* sp. WK-3419,^35^ which showed biological activity against HIV-1 replication. Eurystatins A and B (**8** and **9**, respectively, Figure 7), purified from *Streptomyces eurythermus* R353-21,^36^ and the pentapeptide poststatin (**10**, Figure 7), isolated from *Streptomyces viridochromogenes*,^37^ have been shown to inhibit prolyl endopeptidase. It is worth mentioning cyclotheonamides (**11**, Figure 7), a family of macrocyclic pentapeptides isolated from the Japanese marine sponge *Theonella swinhoei*,^38,39^ which manifested potent inhibition of serine proteases. The 2-oxoamide moiety actively takes part in the mechanism of action of these molecules, probably forming a reversible tetrahedral adduct with a hydroxyl group of the enzyme active site (see for example in Figure 8 the X-ray structure of cyclotheonamide A in complex with trypsin).^40^

Among natural products containing the 2-oxoamide functionality, antitumoral properties have been shown by the macrocyclic depsipeptide aplidine (or dehydrodidemnin B **12**, Figure 7), which was isolated in 1990 from the Mediterranean invertebrate *Aplidium albicans*. It is currently under investigation in multiple phase II and III trials for the treatment of different forms of cancer.^41^

In this perspective, we provide a synopsis of some of the applications of the α-ketoamide in drug design, either as a nonelectrophilic or electrophilic moiety. In the former case, the α-ketoamide has been employed for its ability to confer a certain degree of rigidity or flexibility to the molecule and the potential capacity to establish hydrogen bonds with the target biomolecules. In the latter case, the α-ketoamide has been conveniently used for its ability to covalently react through the carbonyl group with catalytic amino acid residues of the target, usually serine or cysteine. These two amino acid residues are extensively exploited as druggable sites for enzyme inhibition, including phospholipases and proteases.

## 2 α-Ketoamide as a Nonelectrophilic Moiety in Potential Drugs

The α-ketoamide moiety has been deeply exploited for its ability to modulate the conformation of lead compounds by increasing or decreasing their structural rigidity or by conferring the capacity to establish hydrogen bonds, in order to improve their potency and pharmacokinetic profile and thus broaden their potential use as pharmacological tools.

### 2.1 Benzodiazepine Receptor (BzR) Ligands

The α-ketoamide frame with its potential to assume a pseudoplanar disposition and engage in a noncovalent interaction was employed with the aim of developing novel ligands for the benzodiazepine receptor (BzR), a binding site by which the benzodiazepines (Bzs) exert their pharmacological actions.^44,45^ This site is situated at the interface of the α and γ subunits of the type A receptor of the γ-aminobutyric acid (GABA_A_), the main inhibitory neurotransmitter in the central nervous system. BzR ligands allosterically modulate the affinity of GABA for its binding site, spanning from agonists (with anxiolytic, anticonvulsant, sedative-hypnotic, and myorelaxant functions) through antagonists to inverse agonists (with anxiogenic, proconvulsant, or even convulsant activities). The majority of Bz-sensitive GABA_A_ receptor subtypes in the brain are α_1_β_3_γ_2_ (mediating sedation), α_2_β_3_γ_2_ (mediating anxiolysis and myorelaxation), α_3_β_3_γ_2_(mediating anxiolysis), and α_5_β_3_γ_2_ (associated with cognition, learning, and memorizing), while the α_4_β_3_γ_2_ and α_6_β_3_γ_2_ subtypes are called Bz-insensitive receptors because they do not respond to Bzs. The α subunit regulates affinity and efficacy of BzR ligands, differently from the γ_2_ and the β subunits.^46,47^

Starting from the late 1980s, extensive research programs focused on the development of new compounds with different affinities, efficacies, and selectivities for the various GABA_A_/BzR-subtypes. Structure–activity relationships (SARs) of structurally different classes of BzR ligands were rationalized in light of a pharmacophore/topological receptor model made up of a hydrogen bond acceptor (A_2_), two hydrogen bond donors (H_1_ and H_2_), four lipophilic regions (L_1_, L_2_, L_3_, and L_Di_), and three sterically forbidden sites (S_1_, S_2_, and S_3_).^48^ In all cases, only planar or pseudoplanar compounds were capable of effectively interacting with the binding site.

**Figure 9:**
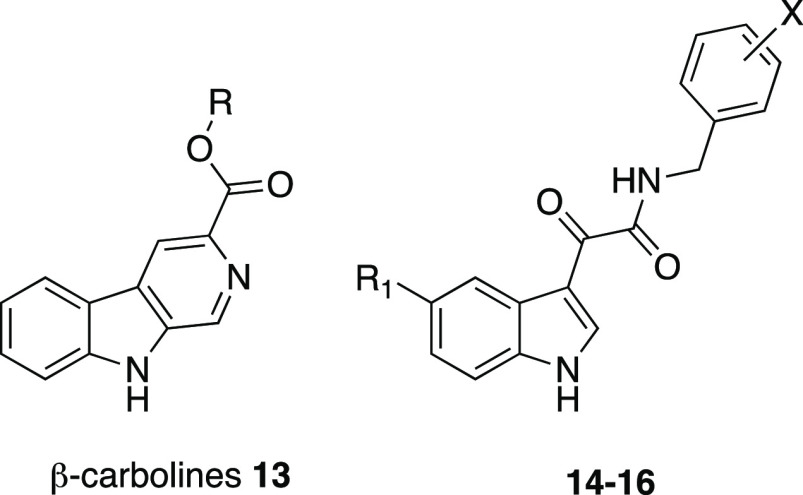
Structures of β-carbolines **13**(49) and indol-3-ylglyoxylamides **14**–**16**.^44^

In this context, the α-ketoamide moiety, being able to assume a pseudoplanar conformation if conjugated with an aromatic system, was exploited by Martini et al. with the aim of developing novel BzR ligands.^44^ A number of *N*-(substituted)-indol-3-ylglyoxylamides **14**–**16** were developed^44^ as “open-ring” analogues of β-carbolines **13** (Figure 9), a class of high affinity BzR ligands; in compounds **14**–**16**, the C=O distal from the indole mimics the *N*-atom of the carboline and the α-ketoamide should be able to maintain the planar spatial disposition of parent compounds **13**.^49^

**Figure 10:**
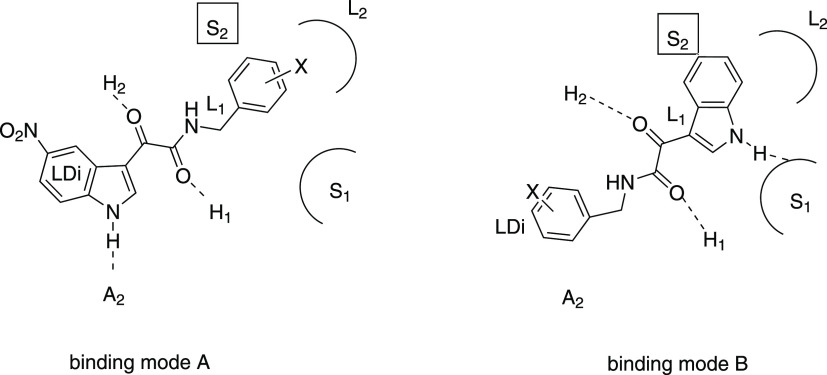
Binding modes A and B of indoles^51^ within Cook’s pharmacophore/topological model.^48^

Compounds featuring a variously substituted benzyl group at the amide nitrogen showed higher affinity for the α_1_ with respect to the α_2_ and α_5_ BzR isoforms (see Table 1 for representative compounds **14**–**16**).^50−52^ Data indicated interdependent effects of the R_1_ and X substituents on α_1_ affinity, suggesting that these compounds might interact with the receptor, adopting two different binding modes shown as A and B in Figure 10 for two representative benzylamino-derivatives.^51^

**Table 1:**
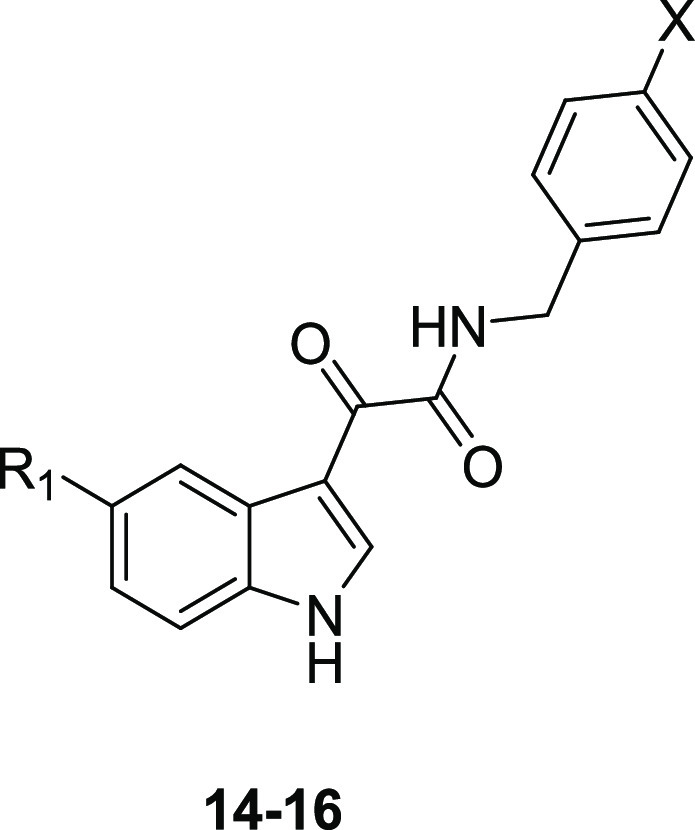
BzR Affinity for Compounds **14**–**16**^a^

			*K*_i_ (nM) or % inhibition (10 μM)^b^^,^^c^			
cpd	R_1_	X	bovine brain membranes	α_1_β_2_γ_2_	α_2_β_2_γ_2_	α_5_β_3_γ_2_
**14**^c^	H	H	120 ± 11	346 ± 29	39% ± 3	46% ± 5
**15**^d^	NO_2_	H	117 ± 12	65 ± 5	32% ± 3	44% ± 4
**16**^e^	NO_2_	CH_3_	88 ± 6	31.3 ± 2	0%	0%
diazepam			10 ± 1			
flumazenil			0.90 ± 0.05			
clonazepam			0.85 ± 0.02			

a *K*_i_ (nM) or % inhibition (10 μM) data of indol-3-ylglyoxylylamide derivatives **14**–**16**.

b *K*_i_ represents the means ± SEM of three determinations performed in triplicate.

c Inhibition percentages of specific [^3^H]-flumazenil binding at 10 μM represent the means ± SEM of three determinations performed in triplicate.

d Data are from Da Settimo et al.^50^

e Data are from Primofiore et al.^52^

In both binding modes, the α-ketoamide with its oxygen atoms of the CO1 and CO2 is hydrogen-bonded to the H_2_ and H_1_ sites. The two binding modes differ in the other interactions. Briefly, in mode A, the indole NH engages in an interaction with the A_2_ site and the L_1_, L_2_, and L_Di_ lipophilic pockets are occupied by the CH_2_, the phenyl, and the fused benzene, respectively; the presence of an electron-withdrawing substituent at the 5-position (Cl or NO_2_) produces a beneficial effect on affinity as it reinforces the NH···A_2_ hydrogen bond. In mode B, the indole nucleus occupies the lipophilic L_1_ and L_2_ regions, and the indole NH hydrogen bonds to a heteroatom of the S_1_ site. Only 5-unsubstituted indoles may adopt this binding mode because the S_2_ site closely faces the indole 5-position. A large number of variously substituted indol-3-ylglyoxylamides were prepared and tested as BzR ligands with the aim of obtaining affinity-based selectivity throughout the different BzR isoforms. Various literature reports indicated that the L_2_ and L_Di_ regions might play a crucial role in conferring ligands’ selectivity as they differ in dimensions in the various subtypes: (i) L_Di_ and L_2_ pockets are larger in the α_1_ and α_5_ isoforms, respectively, and, consequently, their full occupation may lead to α_1_ and α_5_ selective compounds, respectively;^53^ (ii) the concomitant occupation of L_2_ and L_Di_ may produce α_2_ selectivity;^54^ (iii) a potent interaction with the L_Di_ pocket, despite occupation of other lipophilic areas, may lead to α_1_ selective compounds.^53^ On the basis of these findings and taking into account the hypothetical binding modes of indole BzR ligands (Figure 10), a library of *N*-substituted indol-3-ylglyoxylamides able to fill the L_Di_ and L_2_ pockets differently was investigated.^52^ All ligands show fair to high α_1_ selectivity affinity with respect to α_2_ and α_5_ subtypes, regardless of the interaction with the L_1_/L_2_ regions, reasonably due to their strong interaction with the L_Di_ pocket, as reported in the literature for other series of potent BzR ligands.^53,55,56^ Compound **16** was identified as an affinity-based α_1_-selective ligand (*K*_i_ 31.3 nM) and evaluated in a functional assay resulting in a full agonist at the α_1_ subtype.^52^ In addition, when assayed in a behavioral model based on the examination of the spontaneous motor activity of mice, compound **16** has proven to be a sedative-hypnotic agent, although less active than the reference zolpidem.^52^

**Figure 11:**
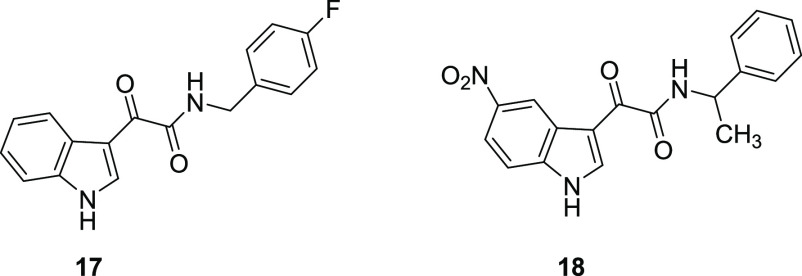
Structures of anxioselective indolyl-2-ketoamides **17** and **18**.^58^

Anxioselective agents may be identified among compounds binding selectively to the α_2_β_*x*_γ_2_ subtype of the GABA_A_/BzR complex and behaving as agonists or among compounds binding with comparable potency to various BzR subtypes but eliciting agonism only at the α_2_β_*x*_γ_2_ receptor. Because of subtle steric differences among BzR subtypes, the latter approach has proved much more successful. Compared to classical nonspecific Bzs, either affinity- or efficacy-based α_2_ selective agonists should maintain anxiolytic activity without unwanted side effects such as sedation, tolerance, dependence, and cognitive processes impairment.^46^ In this connection, the same research group investigated some indol-3-ylglyoxylamides of their in-house library for the potential as anxioselective agents,^51,52,57,58^ identifying, as the major result, compounds **17** and **18** (Figure 11) as α_2_ functionally selective agonists producing anxioselective/not sedating effects in vivo.^58^

**Figure 12:**
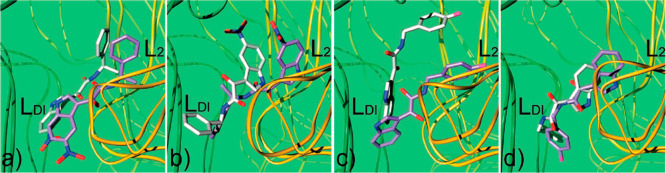
Binding conformations of **18** (a and b) and **17** (c and d) in pose **A** (a and c) and **B** (b and d) into the BzR cleft. The receptor is represented as green (γ subunit) and orange (R subunit) ribbons. Ligands in their docked conformations are represented in purple sticks, while ligands in conformations calculated through MD simulations are represented as white sticks.^58^ Reproduced from ref (58). Copyright 2009 American Chemical Society.

The crucial role played by the α-ketoamide in the interaction of these compounds with the target protein was confirmed by molecular modeling studies (Figure 12). Results from these studies are in agreement with the previously formulated hypothesis according to which two binding modes are possible for these ligands in which the α-ketoamide establishes a double H-bond with the H_1_ and H_2_ donor sites, while indole and phenyl rings can be alternatively accommodated in the L_2_ and L_Di_ pockets (see Figure 10).^58^ The binary complexes calculated by the docking program for compounds **17** and **18**, in both modes A and B, were also subjected to molecular dynamic (MD) simulations to refine the predicted binding geometries. Results suggested that the presence of the 5-nitro group in **18** would allow for the formation of more productive interactions when the indole is lodged in the L_Di_ pocket (binding mode A), while, for unsubstituted compounds like **17**, the binding mode B is more reasonable (Figure 12).

### 2.2 Translocator Protein (TSPO) Ligands

The nonreactive α-ketoamide has been employed by Da Settimo et al.^59−61^ to develop new anxiolytic agents with improved safety profiles, targeting the translocator protein (TSPO),^45,62,63^ a 18 kDa mitochondrial protein which facilitates the transport of cholesterol into mitochondria,^64^ where it is converted into pregnenolone, the precursor of endogenous steroids.^65^ Neurosteroids positively modulate GABA neurotransmission by interacting with a specific site on the GABA_A_ complex that is distinct from that of Bzs and produce nonsedative anxiolytic effects.^62^ Thus, neurosteroidogenic TSPO ligands are considered a viable alternative for the treatment of anxiety, without the typical side effects correlated to Bzs.^66,67^

**Figure 13:**
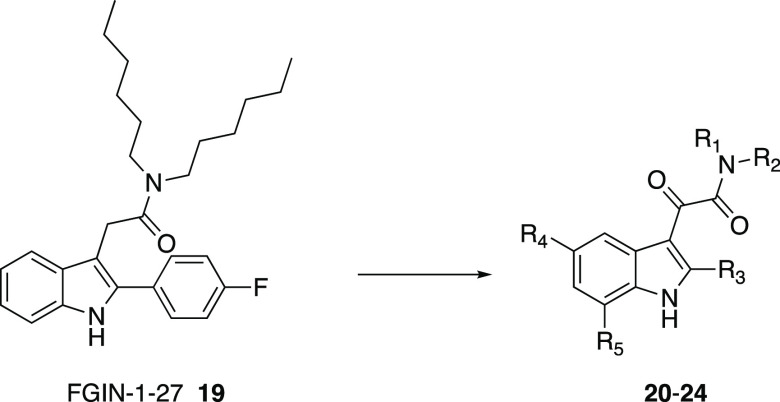
Structures of TSPO ligands FGIN-1–27 **19**,^68^ and *N*,*N*-dialkyl-2-arylindol-3-ylglyoxylamides **20**–**24**.^61^

In this context, the authors employed the α-ketoamide motif to constrain the structural flexibility of the 2-arylindol-3-acetamides, e.g., FGIN-1-27 (**19**), described by Kozikowski et al. as TSPO selective high affinity ligands (Figure 13)^68^ that are structurally similar to the indolylglyoxylamides previously reported as BzR ligands.^52,58^ A wide library of *N,N*-dialkyl-2-arylindol-3-ylglyoxylamides was developed (**20**–**24**, Figure 13); many compounds showed high TSPO affinity with *K*_i_ values in the nanomolar/sub-nanomolar range and complete selectivity for TSPO versus BzR (Table 2).^59−61^

**Table 2:**
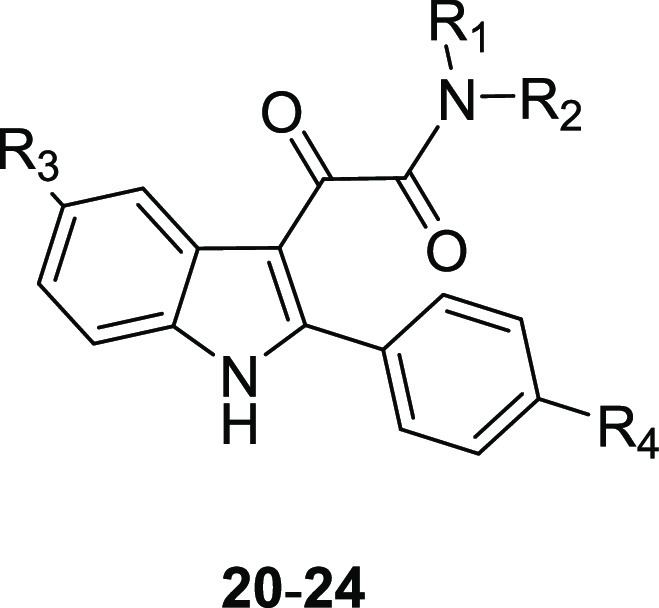
TSPO Affinity of Compounds **20**–**24** and Their Effects on Increase of Pregnenolone Production

cpd	R_1_	R_2_	R_3_	R_4_	*K*_i_ (nM) or inhibition (%)^a^	increase of pregnenolone production vs control (%)^b^
**20**^c^	(CH_2_)_5_CH_3_	(CH_2_)_5_CH_3_	Cl	Cl	5.8 ± 0.6	166 ± 12
**21**^d^	CH_2_CH_3_	CH_2_C_6_H_5_	Cl	Cl	3.33 ± 0.3	171 ± 14
**22**^c^	(CH_2_)_3_CH_3_	(CH_2_)_3_CH_3_	Cl	Cl	1.9 ± 0.2	147 ± 13
**23**^d^	(CH_2_)_5_CH_3_	(CH_2_)_5_CH_3_	F	Cl	7.75 ± 1.55	135 ± 4
**24**^c^	(CH_2_)_2_CH_3_	(CH_2_)_2_CH_3_	CH_3_	H	5.50 ± 0.38	148 ± 12
**Ro5-4864**					23 ± 3.1	41 ± 4
**PK11195**					9.3 ± 0.5	48 ± 5

a The concentration of compounds that inhibited [^3^H]PK11195 binding in rat kidney mitochondrial membranes (IC_50_) by 50% was determined with six concentrations of the displacers, each performed in triplicate. *K*_i_ values represent the mean ± SEM of three determinations.

b C_6_ glioma cells were incubated for 2 h at 37 °C in the presence of each compound. Pregnenolone was measured by radioimmunoassay. Values are the mean ± SEM of three determinations.

c Data taken from Primofiore et al.^59^

d Data taken from Da Settimo et al.^60^

Noticeably, the indolyl-2-ketoamides displayed a gain in TSPO affinity of at least 1 order of magnitude when compared to their indolyl-3-acetamide counterparts. Several of the most potent 2-aryl-indolylglyoxylamides were also able to enhance pregnenolone production in rat C6 glioma cells (Table 2).^59−61^ Docking studies were performed on this class of compounds, and the proposed binding mode evidenced that the presence of the α-ketoamide moiety, rather than establishing specific interactions with the receptor, plays a crucial role in constraining the flexibility of the ligand branch, allowing the ligand to assume the bioactive conformation.^61^

To correlate the ability of ligands to enhance neurosteroid production in vitro with potential anxiolytic effects in vivo, compounds **21** and **24** (30 mg/kg, i.p.) were evaluated in a rat anxiety model, evidencing an anxiolytic-like effect, without any sedative activity.^60,69^

However, as for many classes of TSPO ligands reported in the literature, no correlation between TSPO affinity and in vitro efficacy was observed for this class of compounds. This issue limits the identification of lead compounds by means of the traditional affinity-based drug discovery processes and also questions about the specificity of the biopharmacological effects observed.^70,71^

Recently, it has been demonstrated that the “residence time” (RT), defined as the time spent by the ligand bound to its target, is more accountable for the determination of in vitro effects of a molecule, rather than its affinity for the target.^72^ For these reasons, some 2-arylindolylglyoxylamide TSPO ligands^59−61^ were selected on the basis of their different abilities to stimulate in vitro steroidogenesis and their RTs were quantified.^73^ Obtained data indicated that the ability of compounds to stimulate steroidogenesis positively correlated with their RT. A positive relationship between RT and in vivo anxiolytic activity for three compounds was also observed, demonstrating that RT plays a determinant role not only in the in vitro steroidogenic efficacy but also in the in vivo anxiolytic effect of new TSPO ligands.^73−76^

Very recently, the same research group set up an enhanced-sampling MD protocol that allowed them to unravel the structural reasons for different RTs of 2-arylindol-3-ylglyoxylamides with a similarly high TSPO affinity. The ligands’ dissociation paths were studied, and the results suggested that subtle structural differences have a substantial effect on the dissociation energetics: slowly dissociating compounds were able to establish tight interactions within a specific region of the protein, different from the rapidly dissociating ones. Interestingly, in vivo studies further support these findings, evidencing how the anxiolytic effect observed for the 2-arylindol-3-ylglyoxylamides correlates with their RT to TSPO.^77^

Shortly thereafter, this class was further investigated by the same research group in order to develop compounds potentially useful for a different therapeutic application, that is, the multitarget therapy against glioblastoma multiforme (GBM), a particularly aggressive form of brain cancer.^45^ Multitarget therapy offers many advantages compared to monotherapy in several diseases, including cancer, since targeting different pathways leads to an increase of the therapeutic effectiveness and tolerability and a decrease in drug resistance.^78^ In this context, a series of indolylglyoxyldipeptides was rationally designed to activate TSPO^79^ and the tumor suppressor protein p53,^80,81^ two attractive intracellular targets in GBM treatment, as they play an important role in inducing permeabilization of the outer mitochondrial membrane that triggers mitochondria-mediated cell apoptosis. p53 is one of the most frequently altered proteins in human cancer, and its deregulation is mainly due to the overexpression of its negative regulator, murine double minute 2 (MDM2). Therefore, the MDM2/p53 interaction inhibition represents a viable approach in GBM therapy.^80^

**Figure 14:**
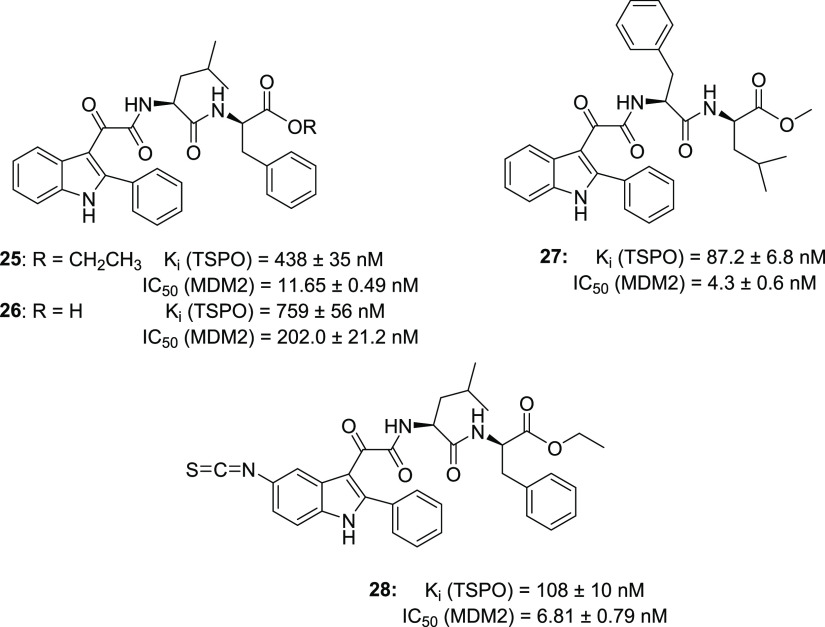
Structures and biological activities of dual TSPO/MDM2 modulators **25**–**28**.^81−83^

Considering the mode of interaction of p53 with MDM2, constituted by a hot spot of three critical residues, namely, Trp23, Leu26, and Phe19, a synthetic molecule displaying three hydrophobic groups in an orientation that mimics these residues could occupy the MDM2 cleft and thereby inhibit the p53-MDM2 binding. Thus, with the aim to rationally design and synthesize dual (TSPO and p53) targeting molecules, the basic structure of the phenylindolylglyoxylamide TSPO ligands,^59−61^ was functionalized with the dipeptide Leu-Phe (**25**, **26**, Figure 14) in order to obtain compounds able to reactivate p53, while retaining TSPO affinity. The phenylindolylglyoxylamide, leucine, and phenylalanine in derivatives **25** and **26** mimic the above-described critical residues Trp23, Leu26, and Phe19. In addition, the presence of the glyoxylamide moiety instead of a peptide element could also confer a greater molecular stability to such compounds. The results clearly showed the ability of **25** and **26** to bind to TSPO (*K*_i_ values of 438 ± 35 nM and 759 ± 56 nM, respectively) and to reactivate p53 functionality by inhibiting its interaction with MDM2 (IC_50_ values of 11.65 ± 0.49 nM and 202.0 ± 21.2 nM, respectively). In GBM cells, both molecules caused mitochondrial membrane potential (Δψm) dissipation and cell viability inhibition, with higher potency compared to the single target reference standards (PK11195 for TSPO and nutlin-3 for p53-MDM2)^80^ singularly applied, due to the synergism resulting from the simultaneous modulation of both targets.

Building on these promising results, the same researchers performed a lead optimization process in a subsequent study by developing a series of derivatives bearing several different dipeptide moieties on the glyoxylyl bridge.^82^

Compound **27** (Figure 14) emerged as the most potent derivative in inhibiting the interaction between p53 and MDM2 with an IC_50_ value of 4.3 ± 0.6 nM and binding to TSPO with a *K*_i_ of 87.2 ± 6.8 nM. **27** was able to restore normal p53 activity and inhibit cell growth of GBM cells through cell cycle arrest and apoptosis. Furthermore, **27** did not affect the viability of a GBM cell line expressing mutant p53, while it was able to impair the proliferation of glioma cancer stem cells (CSCs), that are resistant to therapies and responsible for GBM recurrence. In addition, compound **27** was shown to preferentially direct its antiproliferative effect toward tumor cells compared to healthy ones.^82^

**Figure 15:**
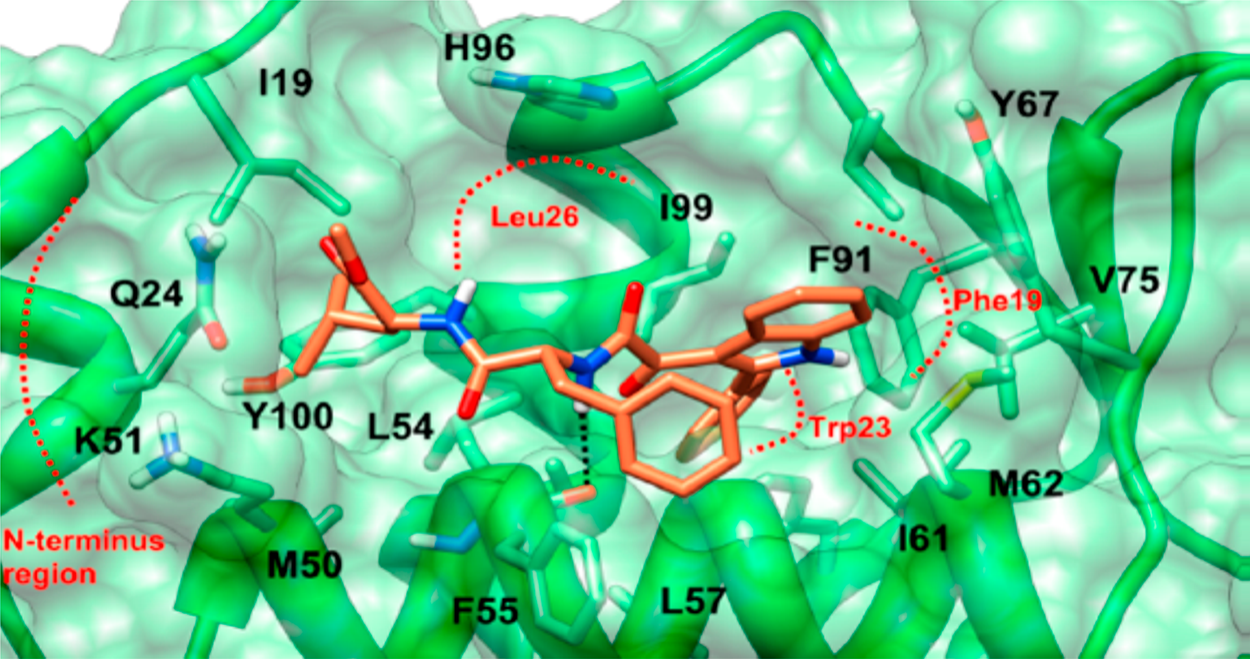
Docking pose of compound **27** in the MDM2 binding cleft. The ligand is shown as coral sticks, the protein surface as transparent green, and the interacting residues as light-green sticks. MDM2 binding pockets are defined in red dots and labeled in accordance with the p53 interacting side chains.^82^ Reproduced from ref (82). Copyright 2016 American Chemical Society.

Finally, with the aim to explain at the molecular level the binding of **27** to MDM2 protein, docking studies were performed evidencing the presence of a H-bond between the glyoxylamide-NH and the backbone carbonyl group of the residue of L54, highlighting the crucial role of this moiety for the interaction with the target protein (Figure 15).

As a continuation of this project, considering that reversible drugs may be ineffective in maintaining their therapeutic effect over time and so favoring the activation of alternative signaling pathways able to escape drug action and cause resistance, a dual target molecule based on the structure of the 2-phenylindol-3-ylglyoxyldipeptide derivative **25** was developed (**28**, Figure 14),^83,84^ characterized by a long-lasting binding profile to TSPO and MDM2. Compound **28**, featuring a 5-isothiocyanate group able to covalently bind SH or NH groups of the target protein, binds TSPO and MDM2 in a covalent manner, with *K*_i_ values of 108 ± 10 nM, and IC_50_ 6.81 ± 0.79 nM, respectively, and inhibits GBM cell growth by causing cell cycle arrest and apoptosis. All these effects seemed to be greater and more long-lasting than those observed for the reversible analogue **25**, evidencing that the dual-targeting irreversible ligand **28** represents an interesting alternative to overcome the time-limited effects of traditional chemotherapies for GBM.^83^

### 2.3 Quorum Sensing (QS) Inhibitors

In the field of the development of antibacterial agents, the α-ketoamide moiety plays a significant role for its noncovalent interaction for quorum sensing (QS) inhibition, that in turn may induce an antibacterial effect. QS is a chemical-mediated mechanism by which bacteria cooperatively regulate various virulence phenotypes, such as the formation of biofilms. The chemical entities that mediate the QS system are called autoinducer. Recently, quorum sensing inhibitors (QSIs) have become potential tools for overcoming antibiotic resistance.^85^ An *N*-acyl homoserine lactone (AHL)-mediated QS system is used by many Gram-negative bacteria. The LuxI/LuxR (expressed in *V. fischeri*) and LasI/LasR (expressed in *P. aeruginosa*) systems are the proteins responsible for the synthesis and recognition of various autoinducers.^86^ However, AHL-based QSIs are sensitive to both nonenzymatic hydrolysis and degradation by lactonases, leading to ring-opened products, which usually lack biological activity. For these reasons, several non-AHL-based QSIs have recently been developed.^87^

**Figure 16:**
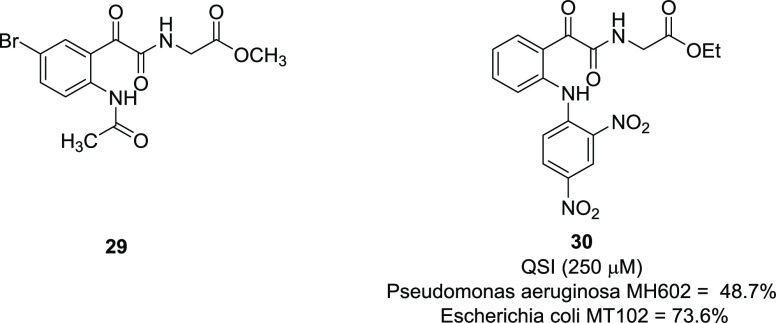
Structures of quorum sensing inhibitors **29** and **30**, biological activity of **30**.^88,89^

Within this context, in virtue of the ability of the peptidomimetics to mimic the properties of natural peptides and to confer greater molecular stability and improved biological activity, Kumar et al. made use of the glyoxylamide moiety to develop a series of novel peptidomimetics as QSIs.^88^ The glyoxamide moiety offers enhanced ability to engage hydrogen bonds, favoring the interactions of such compounds with the LasR receptor protein and therefore compounds’ QS inhibitory activity. The most active compound of the whole series, **29**, is presented in Figure 16.^88^ More recently, the same research group synthesized a new series of *N*-arylisatin-based glyoxamide derivatives, conceived by the ring-opening reaction of *N*-arylisatins, among which **30** (Figure 16) showed the highest QSI activity of 48.7% and 73.6% at 250 μM concentration in *Pseudomonas aeruginosa* MH602 and *Escherichia coli* MT102, respectively.^89^

**Figure 17:**
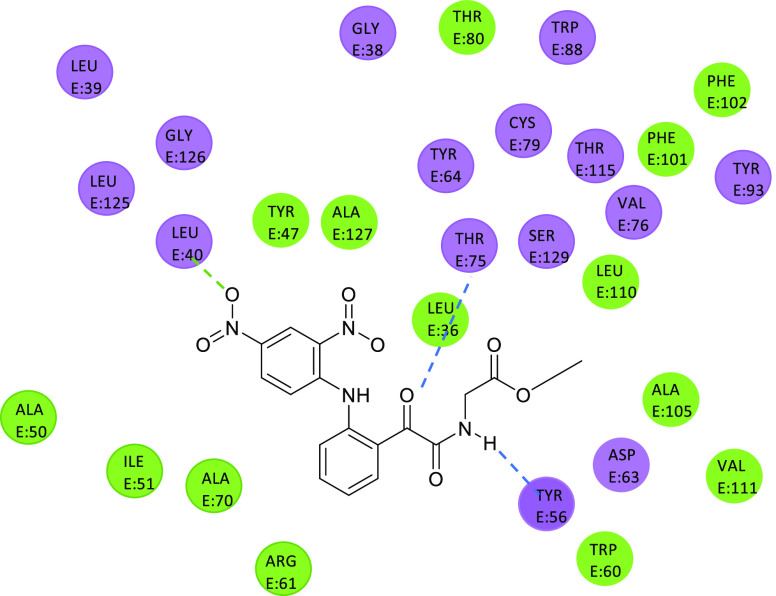
2D-representation of the highest scoring pose of compound **30** with LasR protein; blue dashed lines represent the hydrogen bonds between LasR residues (purple and green) and compound **30**. The green dashed line represents a hydrogen bond between compound **30** and the amide backbone of the LasR receptor.^89^

Docking studies on this class of compounds performed on the LasR receptor protein of *Pseudomonas aeruginosa* evidenced the crucial role played by the formation of a hydrogen bonding network involving the α-ketoamide. Specifically, two hydrogen bonds were proposed, one between a threonine residue (Thr75) and the α-carbonyl group of the oxalyl bridge and one between a tyrosine residue (Tyr56) and the NH glyoxamide (see Figure 17 for representative compound **30**).^89^

### 2.4 Small Molecular Antimicrobial Peptidomimics (SMAMP Mimics)

The nonreactive α-ketoamide has been employed to obtain small molecular antimicrobial peptidomimics (SMAMP mimics) with the aim to overcome the limitations associated with antimicrobial peptides (AMPs), namely, susceptibility to degradation by proteases or peptidases, in vivo toxicity, and nonselective action on microbial strains.^90,91^

**Figure 18:**
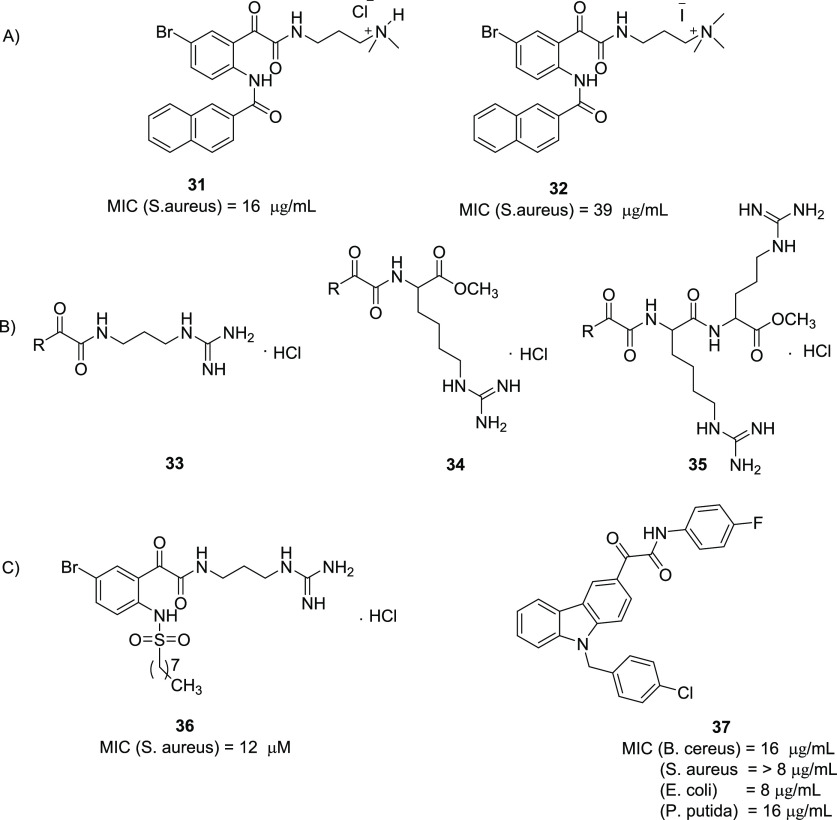
Structures and biological activities of SMAMP mimics **31**–**37**.^90,93−95^

In 2016 Kumar et al., considering the similarity of *N*-phenylglyoxylamides to peptide bonds, synthesized a library of glyoxamides via the ring opening reaction of *N*-naphthoyl-, *N*-benzoyl-, and *N*-hexanoyl-isatins to obtain SMAMP mimics.^90^ In general, derivatives featuring the *N*-benzoyl and *N*-hexanoyl groups did not have significant antimicrobial activity, while all the *N*-naphthoyl-glyoxamides showed good to excellent antibacterial activity against *S. aureus*. Thanks to the AMPs amphipathic in nature,^92^ all compounds were also converted in their corresponding hydrochloric acid and quaternary ammonium iodide salts, causing an increase of antibacterial activity by 2–20 fold. Within this class, compound **31** (Figure 18A) showed the highest antimicrobial activity with a minimum inhibitory activity (MIC) of 16 μg/mL, while the corresponding quaternary ammonium iodide salt **32** (Figure 18A) exhibited good activity with MIC of 39 μg/mL.^90^ Moreover, these derivatives showed a capacity to disrupt established biofilm in *S. aureus*, with compound **31** showing 50%, while compound **32** 46% of disruption of established biofilm at 250 μM. Of note, quaternary ammonium salts are nontoxic to mammalian cells and selectively toxic toward bacterial cells.^90^

The same research group synthesized three novel series of guanidine-embedded glyoxamides via ring opening reaction of *N*-naphthoylisatins, being the guanidine represented in various natural products, antibiotics, and synthetic peptidomimetics with high antimicrobial activity:^93^ (i) in the first series, the quaternary ammonium moiety was replaced by a guanidinium one (**33**, Figure 18B); (ii) the second series was characterized by a guanidyl-lysine moiety (**34**, Figure 18B); (iii) in the third series, an arginine residue was coupled to the terminal lysine residue of **34** (**35**, Figure 18B).^93^ Compounds **33** exhibited moderate to very good antimicrobial activity versus *S. aureus* and lower activity against *E. coli*, while compounds **34** showed lower activity against *S. aureus* but higher activity against *E. coli*. Compounds **35** were the most active. In general, the results showed that the introduction of a guanidinium salt led to compounds with an increased antimicrobial activity with respect to the quaternary ammonium ones. Compounds **35** also showed the greatest levels of biofilm disruption against both Gram-positives (*S. aureus*) and Gram-negatives (*P. aeruginosa*, *S. marcescens* and *E. coli*), and a strongly selectivity profile against bacteria over mammalian cells.^93^

In continuation of the interest in this field, Kumar et al. synthesized a library of *N*-sulfonylphenylglyoxamides.^94^ Among all the investigated compounds, the guanidine derivative hydrochloride **36** (Figure 18C) was shown to be the most promising compound, exhibiting the lowest MIC of 12 μM against *S. aureus*.^94^

The same research group, encouraged by the good antimicrobial activity shown by glyoxamide-based derivatives and by the evidence of the crucial role of carbazole scaffold in bioactive compounds, developed a series of carbazolyl glyoxamides by incorporating these two substructures in a single molecule.^95^ The most promising compound **37** (MIC values ranging between 8 and 16 μg/mL) is presented in Figure 18C.^95^

### 2.5 Antiprion Agents

The α-ketoamide moiety with its ability to form noncovalent interaction was deeply employed in the field of antiprion agents, leading to the generation of highly potent compounds. Prion diseases, or transmissible spongiform encephalopathies (TSEs), are a group of progressive neurodegenerative diseases, which affect both humans and animals. TSEs are associated with the conversion of normal cellular prion protein (PrP^C^) into an insoluble aggregate conformer PrP^Sc^, in which “Sc” stands for scrapie, the prion disease of sheep and goats, that is thought to be infectious. Indeed, these aggregates are suppose to cause death of neuronal cell in TSEs, forming vacuoles and leading to the characteristic spongiform degeneration of brain tissue. Physiological function of PrP^C^ remains widely unclear, but it is highly expressed in neurons and conserved across mammalian species. It appears to play an important role in neuroprotection, cell adhesion, and iron metabolism.^96,97^

**Figure 19:**
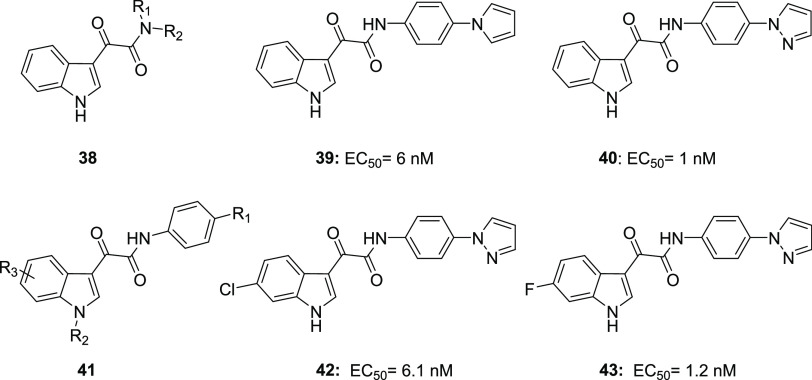
Structures and biological activities of antiprion agents **38**–**43**.^98,100^

Thompson et al. designed and synthesized a wide number of indole-3-glyoxylamides with the general structure **38** (Figure 19). This structure, which emerged from a scrapie-infected mouse brain (SMB) cell line screening assay, was selected after considering the wide variety of drug candidates containing this moiety in various phases of clinical or preclinical studies across a range of biological activities.^98,99^

Testing the compounds for their ability to inhibit PrP^Sc^ formation in a prion infected cell line (SMB) of mesodermal origins revealed that activity in the nanomolar range was achieved only by derivatives featuring at the glyoxylamide position an aniline moiety that is *para*-substituted with an aromatic heterocycle with at least one hydrogen-bond acceptor (**39** and **40**, EC_50_ 6 nM and 1 nM, respectively, Figure 19).^98^

SAR studies at C-4- to C-7-positions about the indole ring (**41**, Figure 19) highlighted that,^100^ whereas derivatization at C-4, C-5, and C-7 was not tolerated, substitution at C-6 proved to be effective in improving the antiprion activity. The presence of strongly electron-withdrawing groups at C-6 represented the best way to obtain compounds with an optimal antiprion effect (compounds **42** and **43**, EC_50_ 6.1 nM and 1.2 nM, respectively, Figure 19).^100^ Biological assays on zebrafish performed to better define the toxicity profile of these compounds showed no effect on zebrafish survival for over half of tested molecules, including the most potent candidates. Substitutions at R_1_ with methyl or morpholine should be avoided due to a mortality rate of at least 20%. All the 6-substituted analogues displayed enhanced microsomial stability, suggesting the 6-position as a probable locus of metabolism of unsubstituted molecules.^100^

Thompson et al. developed another series of antiprion agents, first enlarging the set of *p*-substituted indole-3-glyoxylamides and then modifying the glyoxylamide moiety.^101^ This study reconfirmed that the best R_1_ group of indole-3-glyoxylamide derivatives is a 5-membered aromatic ring with at least two heteroatoms. If additional heteroatoms are present, at least one should be oxygen; further modification of the heterocycle is generally detrimental. These results were also confirmed by *in silico* analysis.^101^

**Figure 20:**
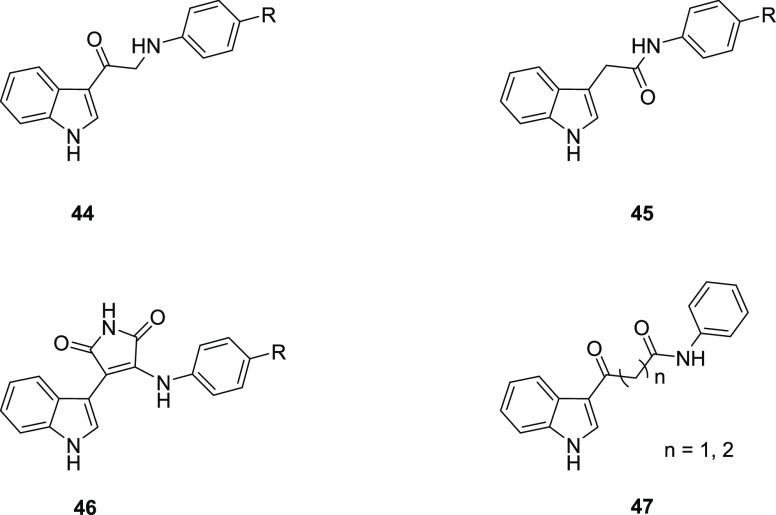
General structures of indole-based derivatives **44**–**47** with modifications at the glyoxamide moiety.^101^

Most importantly, the crucial role of the 2-oxoamide moiety was elucidated through systematic modifications: (1) replacement of either carbonyl by a methylene group, leading to the synthesis of 3-(aminoacetyl)indoles **44** and indole-3-acetamides **45**; (2) substitution with a maleimide bridge **46**; (3) introduction of a one- or two-carbon spacer between the two carbonyls **47** (Figure 20). All the modifications produced a reduction in terms of potency outlining the crucial relevance of the glyoxylamide substructure in order to retain potent antiprion activity. Between the two series that lacked either the amide carbonyl **44** or the α-keto carbonyl **45**, the latter showed a pronounced reduction in activity, suggesting a more substantial role of the carbonyl close to the indole core in conferring potency to the molecules.^101^

### 2.6 HIV-1 Inhibitors

The human immunodeficiency virus (HIV) infection pandemic is now over 25 years old and continues to present a serious health concern for the estimated 37 million people who are infected.^102^ The spread of HIV has been decelerated by highly active antiretroviral therapy (HAART), and, for many infected people, HIV has been transformed into a chronic disease. However, with long-term usage of HAART, some limitations have emerged, such as the onset of resistance. Addressing this problem requires the development of new antiretroviral agents that are able to target different steps of the replication cycle, with improved tolerability and dosing schedules.^103^ A crucial event in HIV infection is the specific interaction between the membrane-bound HIV-1 glycoprotein 120 (gp120) and cluster of differentiation 4 (CD4), the primary attachment receptor for HIV-1. Inhibition of this interaction would likely hamper HIV-1’s infectivity at a very early step of the viral life cycle.^104^

In this context, the glyoxylamide derivative **48** emerged from a cell-based screening assay and was shown to interfere with the gp120/CD4 interaction. An optimization program on **48** yielded compounds strongly able to inhibit HIV-1 infection in vitro,^105−108^ including the glyoxylamide **49** (Table 3) that exhibits nanomolar EC_50_ values (4.0 and 4.9 nM against two different viral strains, CCR5-dependent JRFL and CXCR4-dependent LAI strains of HIV-1, respectively) and no cytotoxicity to the HeLa host cell line.^106^

**Table 3:**
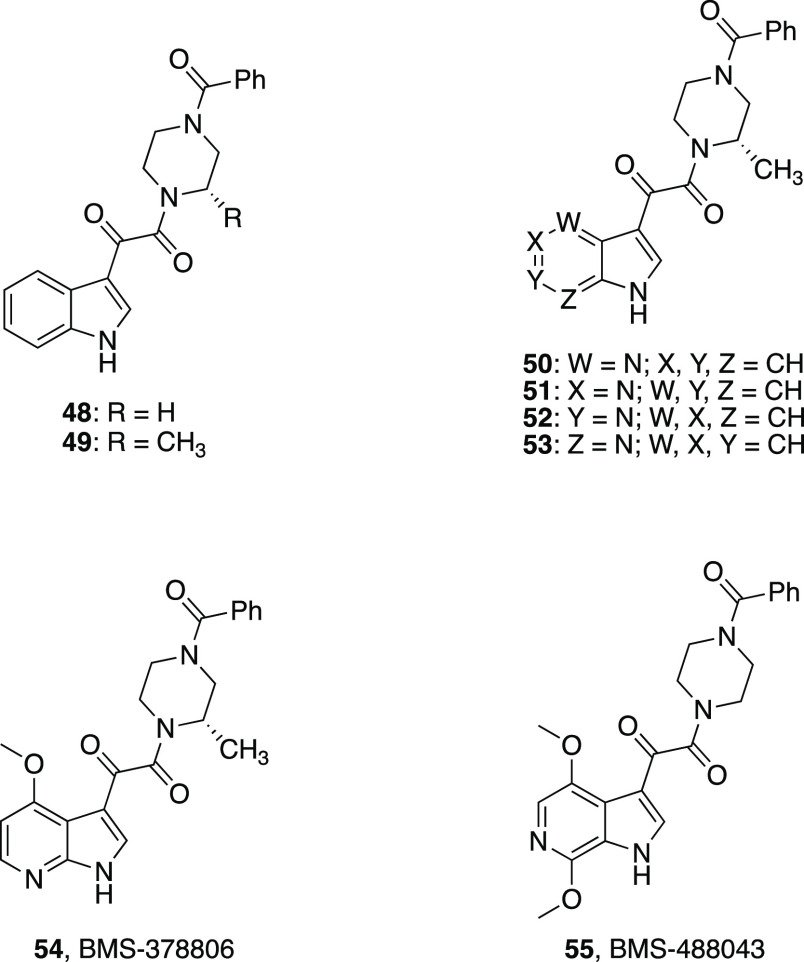
Biological Activity of HIV-1 Attachment Inhibitors **48**–**55**^a^

cpd	EC_50_ [nM]	CC_50_ [μM]
**48**	86 ± 24 (LAI)	145 ± 23
**49**	4.0 (JR-FL)	200
	4.9 (LAI)	
**50**	1.52 (JR-FL)	>300 (*n* = 2)
**51**	575.9 (JR-FL)	>300 (*n* = 2)
**52**	21.6 (JR-FL)	>300 (*n* = 2)
**53**	1.7 ± 1.6 (JR-FL, *n* = 11)	280
**54**	1.47 ± 0.63 (JR-FL)	>300
	2.68 ± 1.64 (LAI)	
**55**	0.88 ± 0.46 (JR-FL, *n* = 56)	>300
	1.15 (LAI)	

a Data are taken from refs (15 and 109).

However, this class of HIV-1 inhibitors presented difficulties associated with their physicochemical properties, giving rise to drug formulation and delivery issues. Several weaknesses emerged in the profile of **49**, mostly the moderate stability in human liver microsomes (HLM) and low aqueous solubility that predict potential problems in preclinical and/or clinical development. In order to solve this problem, which was attributed in part to the properties of indole, four possible azaindole analogues of **49** were synthesized (**50**–**53**, Table 3), all gaining improved pharmacokinetic and pharmaceutical profiles.^109^ The antiviral potency of **49** was maintained for the 4-aza **50** and the 7-aza **53** isomers, whereas incorporation of the nitrogen atom in a less hindered position of the core led to a decrease in HIV-1 inhibitory activity (the 6-aza isomer **52** and the 5-aza analogue **51** were 5- and 100-fold less potent, respectively). All of the isomers **50**–**53** showed an enhanced metabolic stability with respect to **49** (half-life (*t*_1/2_) in HLM: **49** 16.9 min; **50**–**53** from 38.5 to >100 min). The presence of a basic nitrogen atom in the azaindole ring may allow the conversion of the compounds into the corresponding salts, facilitating their formulation.^109^ The increased basicity exhibited by the azaindoles seemed to correlate with their permeability across a Caco-2 monolayer at pH 6.5. The 7-azaindole **53** (p*K*_a_ 2.0) should predominantly be present as a free base, making it highly permeable, while the 4-azaindole **50** (p*K*_a_ 5.0) should exist also in the protonated form, leading to reduced permeability. On the contrary, a large amount of both the 5-azaindole **51** (p*K*_a_ 6.2) and 6-azaindole **52** (p*K*_a_ 6.0) would be present as the pyridinium cation at pH 6.5, reducing the penetration rate across the Caco-2 membrane.

**Figure 21:**
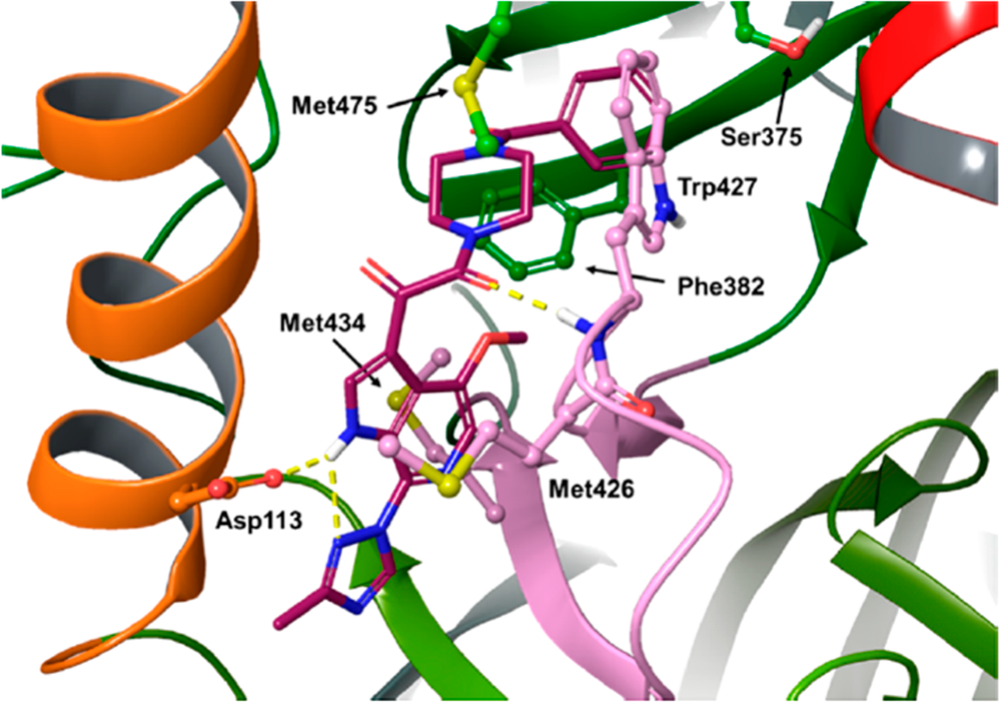
X-ray structure of the cocrystal of the gp120/**56** complex.^15^ Reproduced from ref (15). Copyright 2017 American Chemical Society.

A further optimization of these azaindoles led to the identification of two compounds which advanced to clinical studies: the 7-azaindole HIV-1 attachment inhibitor BMS-378806 (**54**)^110^ and the 6-azaindole derivative BMS-488043 (**55**) (Table 3).^111^ Compound **55** showed an improved in vivo pharmacokinetic profile in rat, dog, and monkey and appeared to address the low permeability and the moderate metabolic stability which represented the most critical drawbacks of **54**, whose development was halted for its low plasma concentration after oral administration in humans (**54**: *t*_1/2_ in HLM 37 min, Caco-2 permeability 51 nm/s; **55**: *t*_1/2_ in HLM 100 min, Caco-2 permeability 178 nm/s).^109,112^ Clinical studies conducted on **55** showed that when administered as monotherapy for 8 days, it reduced viremia in HIV-1-infected subjects, validating the use of HIV-1 inhibitors as potential treatment of HIV-1 infection in vivo.^113^ More recently, starting from compound **48**, an extensive optimization campaign led to the identification of temsavir **56** (GSK2616713, Table 4), which showed enhanced antiviral activity against a spectrum of laboratory strains (Table 4) and good pharmacokinetics (PK).^15^ Mechanistic studies relying on X-ray structure of crystal complex **56**/gp120 evidenced the ability of such compounds to bind to gp120 at the interface between the inner and outer domains under the β20−β21 loop (Figure 21).^114^ Despite a predominance of hydrophobic interactions, H-bonds were observed between the backbone NH of W427 with the oxoamide carbonyl and the azaindole NH and the side chain of D113. The benzamide occupies the gp120 site that is also occupied by W427, so that W427 and the β20−β21 loop are pushed toward the CD4 binding loop, resulting in the inhibition of CD4 binding (Figure 21).^15^

**Table 4:**
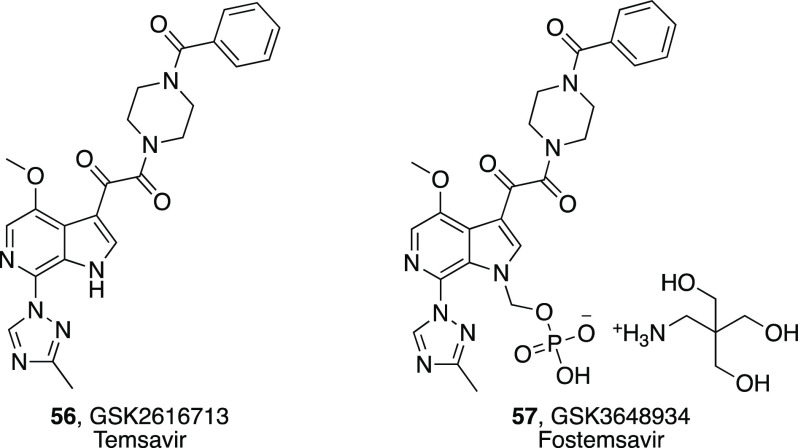
Activity in Vitro of **56** against Laboratory Strains of HIV-1^a^

coreceptor tropism	virus	EC_50_ [nM]
**CCR5**	JR-FL	0.4 ± 0.1
	SF-162	0.5 ± 0.2
	Bal	1.7 ± 0.5
**CXCR4**	LAI	0.7 ± 0.4
	NL4-3	2.2 ± 0.6
	MN	14.8 ± 5.2
	IIIb	16.2 ± 1.7
	RF	>2000

a Data are taken from Meanwell et al.^15^

To solve emerging problems linked to dissolution and solubility-limited absorption, fostemsavir **57** (GSK3648934, Table 4) was synthesized as the phosphonooxymethyl prodrug of **56**. Recent updates from a phase III clinical trial in patients with limited therapeutic options showed a considerably greater decrease in the viral RNA level in patients receiving **57** compared with those receiving placebo during the first 8 days, with efficacy sustained through 48 weeks.^115^**57** gained approval from Food and Drug Administration in July 2020 for patients with limited treatment options.^116^

### 2.7 Phospholipase A2 Inhibitors

Phospholipases A_2_ (PLA_2_’s) constitute a superfamily of lipolytic enzymes that are responsible for the catalysis of the ester bond hydrolysis at the sn-2 position of glycerophospholipids, which generate free fatty acids, including arachidonic acid and lysophospholipids. There are four predominant types of PLA_2_: the secreted PLA_2_ (sPLA_2_); the cytosolic Ca^2+^-dependent PLA_2_ (cPLA_2_); the cytosolic Ca^2+^-independent PLA_2_ (iPLA_2_); and the PAF-AH (platelet activating factor acetyl hydrolases). The other two types are the lysosomal PLA_2_ (LPLA_2_) and the adipose-PLA_2_ (AdPLA). These enzymes use a catalytic dyad/triad (His/Asp for sPLA_2_; Ser/Asp for cPLA_2_ and iPLA_2_; Ser/His/Asp for PAF-AH and LPLA_2_; His/Cys for AdPLA) in order to perform their function.^117^

Researchers at Lilly published a series of papers regarding indole-based derivatives as GIIA sPLA_2_ (referred to by the authors as human non-pancreatic secretory phospholipase A_2_, hnps-PLA_2_) inhibitors.^118^ High levels of GIIA sPLA_2_ are associated with numerous disease states, including acute pancreatitis,^119^ adult respiratory distress syndrome (ARDS), bacterial peritonitis, and septic shock.^120^ Potent and selective GIIA sPLA2 inhibitors would be useful pharmacological tools for treating such diseases.

**Figure 22:**
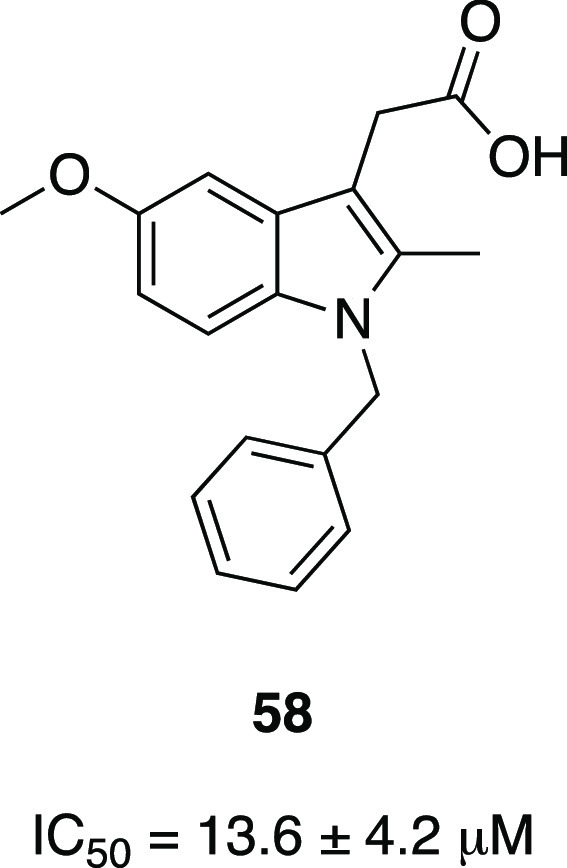
Structure of first indole derivative synthesized by Lilly, **58**.^118^

In this context, an optimization study of the lead compound **58** (IC_50_ 13.6 ± 4.2 μM, Figure 22), obtained by high-volume screening, was performed and included the substitution of the acetate function first with an acetamide moiety and then with the α-ketoamide group.^121^

This last modification proved to be crucial, as exemplified by compounds **59**–**62** (Table 5), in which substitutions at the 4- and 5-position of the indole were also explored, allowing for the optimal potency and selectivity with a 4-oxyacetic acid group to be reached.^122^

**Table 5:**
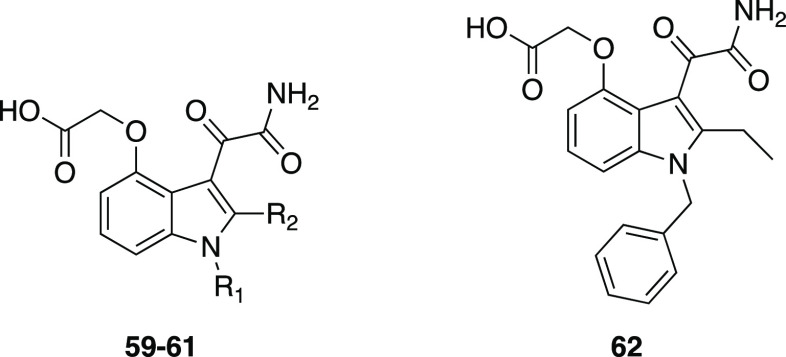
GIIA and GIB PLA_2_ Inhibition by Indole-3-glyoxamides **59**–**62**^a^

cpd	R_1_	R_2_	hGIIA PLA_2_ [μM]	hGIB PLA_2_ [μM]	pGIB PLA_2_ [μM]
**59**	2-(C_6_H_5_)C_6_H_4_CH_2_	CH_3_	0.006 ± 0.001	0.364	0.097
**60**	3-(C_6_H_5_)C_6_H_4_CH_2_	CH_3_	0.009 ± 0.001	0.57	0.007
**61**	C_6_H_5_CH_2_	CH_3_	0.011 ± 0.004	0.761	0.015
**62**			0.009 ± 0.001	0.228	0.048

a Data are taken from Draheim et al.^122^

X-ray crystallography studies confirmed the interaction of the acetamide lead compound **58** with the target protein, also rationalizing efficient binding between the calcium ion in the active site of hGIIA and the two carbonyl groups of compound **62** (LY315920, or varespladib), the carbonyl of the 4-substituent, and the carboxamide carbonyl of the 3-glyoxamide moiety.^123^ Furthermore, the glyoxamide moiety was responsible for novel interactions in the active site, specifically the hydrogen bond between the carboxamide and His48, as well as an interaction between the ketone carbonyl and Phe106 of the enzyme.^122^

Varespladib **62**, also formulated as a methyl ester prodrug, was advanced in several clinical trials for a variety of diseases (i.e., sepsis-induced systemic inflammatory response syndrome, asthma, cardiovascular diseases) but failed in the phase II or phase III due to the lack of efficacy.^124−133^

Inspired by the Lilly research in 1996, a group of researchers from Shionogi reported a series of indolizine and indene derivatives, closely related to the indole-3-glyoxamides as sPLA_2_ inhibitors.^134^ Inhibitory activity was evaluated against recombinant hGIIA PLA_2_ (chromogenic assay) and patient samples (PC/DOC assay);^134^ these data correlated well with SARs found for indole-based derivatives, thus confirming the crucial importance of the synergy between the α-ketoamide moiety at the 3-position and the substituent at 4-position of the central core in coordinating the calcium ion in the active site of the enzyme (**63**–**65**, Table 6). The glyoxamide moiety at different positions was detrimental for the activity, as well as substitutions on the oxoamide nitrogen. Furthermore, the removal of the ketoamide moiety in this series negatively affected the stability to the air and potency against sPLA_2_ (**66**–**69**, Table 6).^134^

**Table 6:**
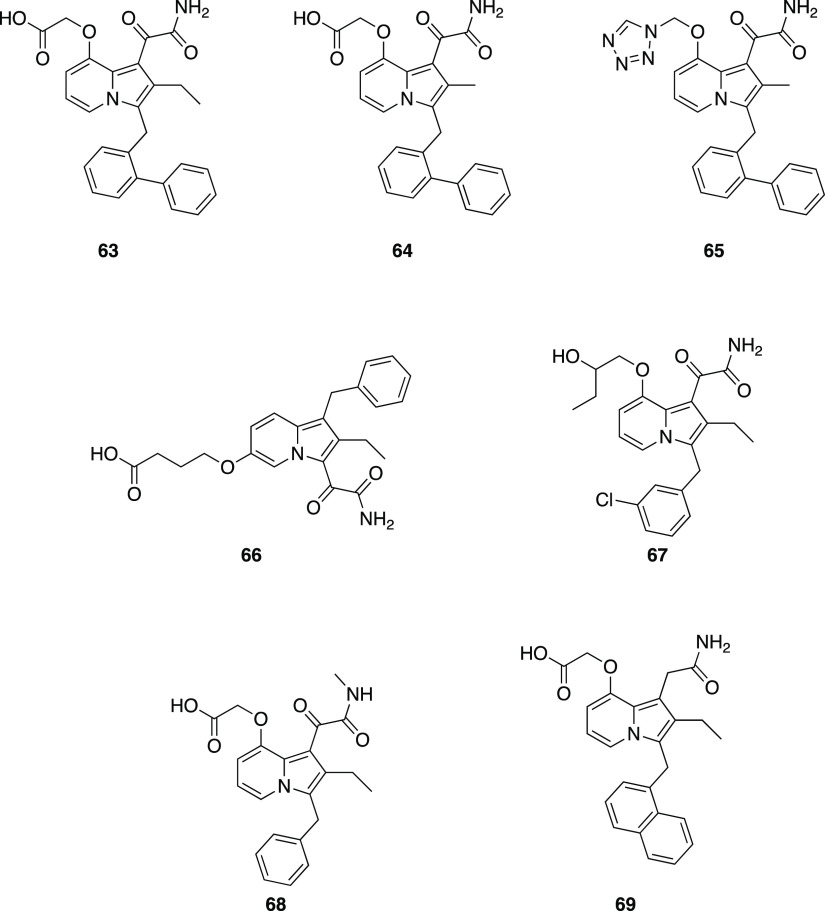
GIIA Inhibition by Indolizine-3-glyoxamide Derivatives^a^

cpd	hGIIA PLA_2_ [μM] chromogenic assay	hGIIA PLA_2_ [μM] PC/DOC assay
**63**	0.006	0.003
**64**	0.008	0.0014
**65**	0.007	0.007
**66**	1.1	b
**67**	19	b
**68**	>50	b
**69**	0.03	0.005

a Data are taken from Hagishita et al.^134^

b Value was not calculated for this compound.

Evaluation of **63**, or indoxam (Table 6), on murine endotoxic shock suggested its capability of blocking the production of proinflammatory cytokines during endotoxemia through PLA_2_-IIA-independent mechanisms, possibly via blockade of the PLA_2_ receptor function.^135^ Compound **64**, called Me-indoxam (Table 6), was found to be the most generally potent sPLA_2_ inhibitor among 12 active site-directed, competitive inhibitors tested on the full set of human and mouse groups I, II, V, X, and XII sPLA_2_’s. The molecule showed potent inhibitory activity toward mGIIA, hGIIA, mGIIC, mGIIE, hGIIE, mGV, and hGV (IC_50_ ≈ 0.01–0.02 μM) and modest inhibitory activity against mGIB, hGIB, mGX, and hGX (IC_50_ ≈ 0.1–1 μM).^136^

On the basis of these findings, researchers further investigated the 3-indole-glyoxamide scaffold as inhibitors of all of the members of the sPLA2; in particular, the group X had the highest specific activity in promoting arachidonic acid release from mammalian cells.^137^ To this end, the authors synthesized a library of 83 derivatives based on crystal structure of **64** with the enzyme, varying the substituent at *N*_1_-position of the indole. The SAR confirmed the necessity of the 3-glyoxamide function together with the 4-(2-oxy-ethanoic acid) moiety and a substituted benzyl group at the *N*_1_-position to gain potency against the sPLA_2_ enzymes, even though no specific selectivity toward sPLA_2_ groups was achieved.^138^

**Figure 23:**
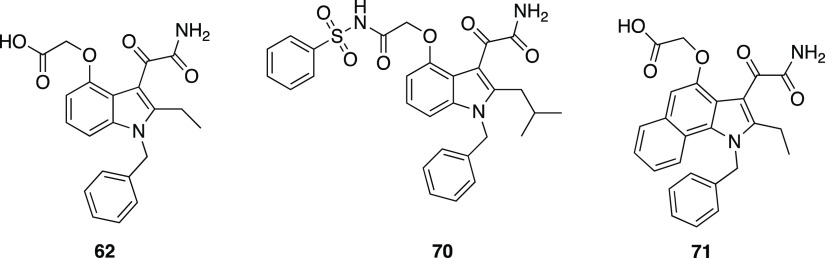
Structures of hGX sPLA_2_ inhibitors **62**, **70**, and **71**.^140^

Varespladib **62** proved to be also a potent inhibitor of the hGX enzyme (IC_50_ 75 nM),^139^ prompting researchers to investigate a series of indole- and indolizine-based derivatives bearing the 2-oxoamide moiety.^140^ Oslund et al. were able to improve potency and selectivity toward the hGX enzyme, replacing the ethyl chain at the 2-position with an isobutyl one and introducing the sulfonamide moiety on the carboxylic function attached at the 4-position of the indole scaffold (**70**, hGX-PLA_2_-IC_50_ 21 ± 7 nM, Figure 23). The benzo-fused analogue (**71**, Figure 23) showed low nanomolar activity values against several human and mouse enzymes and is the most generally potent sPLA_2_ compound to date.^140^

## 3 α-Ketoamide as a Reactive Moiety in Potential Drugs

The ability of the 2-oxoamide moiety to resemble both a scissile amide and ester bond makes it suitable to be included as an electrophilic warhead in designing inhibitors that are analogues of substrates for enzymes responsible for catalyzing the cleavage of those types of chemical bonds through a nucleophilic attack. Particularly, serine and cysteine proteases have been proven over the years to be suitable targets in terms of rational design of novel inhibitors featuring the α-ketoamide moiety. The mechanism of action usually involves the formation of a metastable hemiacetal adduct mimicking the tetrahedral species involved in the catalytic bond cleavage after the nucleophilic addition to the carbonyl group of the inhibitor in the active site.

### 3.1 Serine Proteases

#### 3.1.1 Phospholipase A_2_ Inhibitors

As previously mentioned, PLA_2_s use a catalytic dyad/triad (His/Asp for sPLA_2_; Ser/Asp for cPLA_2_ and iPLA_2_; Ser/His/Asp for PAF-AH and LPLA_2_; His/Cys for AdPLA) to catalyze the hydrolysis of the ester bond at the sn-2 position of glycerophospholipids.^117^ This section will focus on four members of this superfamily of enzymes: GIIA sPLA_2_, GIVA cPLA_2_, GVA sPLA_2_, and GVIA iPLA_2_. GIIA and GVA are part of the secreted phospholipases A_2_ (sPLA_2_), whose involvement in several inflammatory diseases has been described in section 2.7. Studies on cytosolic phospholipase A_2_ (cPLA_2_) GIVA-null mice showed that a reduced production of inflammatory mediators was linked to a better outcome in several pathological conditions such as ischemia-reperfusion injury,^141^ anaphylactic responses,^142^ collagen-induced autoimmune arthritis,^143^ fatty liver damage,^144^ and autoimmune diabetes,^145^ among others, suggesting potential therapeutic uses of inhibitors of this enzyme. Participation of GVIA PLA_2_ in β-cell apoptosis, which may cause the loss of the β-cell mass associated with the onset and progression of type 1 and type 2 diabetes mellitus,^146^ has been suggested by genetically modified mice and cellular studies.^146−149^ GVIA PLA_2_ is responsible also for cardiolipin, a phospholipidic component of the mitochondrial membrane,^150^ deacylation, and monolysocardiolipin accumulation in Barth syndrome,^151^ a disease associated with mutations of the X-linked tafazzin gene (TAZ),^152^ which regulates cardiolipin homeostasis in mitochondria.^153,154^ Accordingly, inhibition of GVIA PLA_2_ could represent a treatment for these pathologies.

**Figure 24:**
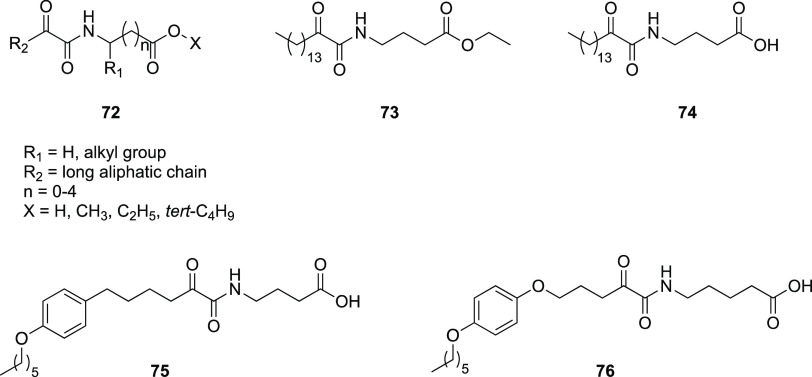
Structures of PLA_2_ inhibitors **72**–**76**.^155−161^

The α-ketoamide warhead has been suitably employed to develop analogues of electrophilic substrates and mimic the tetrahedral species involved in the catalytic cleavage of peptide bonds operated by these enzymes. Kokotos et al. investigated a library of amino acid-based 2-oxoamides as PLA_2_ inhibitors (**72**, Figure 24),^155−159^ outlining the SAR for this class of compounds against the different isozymes. The 2-oxoamide moiety was crucial along with a free carboxyl group for the activity against GIVA cPLA_2_ and GIIA sPLA_2_. Ester variants showed a dual activity against GIVA cPLA_2_ and GVIA iPLA_2_, although with a preference toward the cytosolic phospholipase. The gap between the oxoamide and carboxyl functionalities seems to be correlated with the selectivity against GIVA cPLA_2_ and GIIA sPLA_2_. The cytosolic form appeared to be better inhibited by compounds based on γ- and δ-amino acids, while secreted phospholipase showed more affinity for α-amino acid-based derivatives. All the compounds share a long lipophilic chain which interacts with a hydrophobic region near the catalytic site. Biological results obtained so far were rationalized by a combination of deuterium exchange mass spectrometry (DXMS) and MD simulations for the GIVA cPLA_2_, confirming the model initially proposed by the same group and by molecular docking calculation for GIIA sPLA_2_.^158,159^ A compound from this series, **73** (Figure 24), showed significant affinity for GIVA cPLA_2_ and systemic bioavailability. In addition, **73** resulted in a potent analgesic effect in an in vivo model of centrally and peripherally induced hyperalgesia.^160^

Recently, the same research group investigated the possibility of replacing the long aliphatic chain in order to reduce the lipophilicity of the previously reported 2-oxoamide-based inhibitors (ClogP, ranging from 6.55 to 10.75) that may mean unfavorable ADME properties like poor bioavailability.

A series of analogues of **73** (Figure 24) was synthesized replacing the long aliphatic chain with others bearing an aromatic ring along with one or two ether oxygens. Another strategy they pursued was to incorporate a sulfonamide group or a carboxyl group at the end of the chain to increase polarity. The new compounds were tested against human GIVA cPLA_2_, GVIA iPLA_2_, and GV sPLA_2_.^161^

The importance of the free carboxyl group for selectivity against GIVA cPLA_2_ emerged from these studies. Compound **75** (Figure 24), with the free carboxyl group, presented even better potency toward GIVA cPLA_2_ and showed a molar fraction inhibition value [*X*_I_(50)] of 0.016 associated with diminished lipophilicity (Table 7). Also **76** (Figure 24), bearing two ether oxygens and increased space between the oxoamide functionality and the free carboxyl, presented a *X*_I_(50) value of 0.013 for GIVA cPLA_2_ with reduced lipophilicity (Table 7). Thus, **75** and **76** represent an improvement in comparison to **73** and the corresponding acid **74**, which had *X*_I_(50) values of 0.022 and 0.024, respectively (Table 7). The other attempts to reduce lipophilicity by introducing a sulfonamide moiety or a carboxy group led to inactive molecules.

**Table 7:** Inhibition of GIVA cPLA_2_ by 2-Oxoamides **73**–**76**^a^

cpd	GIVA cPLA2 X_I_ (50)
**73**	0.022 ± 0.009
**74**	0.024 ± 0.015
**75**	0.016 ± 0.004
**76**	0.013 ± 0.002

a Data are taken from Antonopoulou et al.^161^

**Figure 25:**
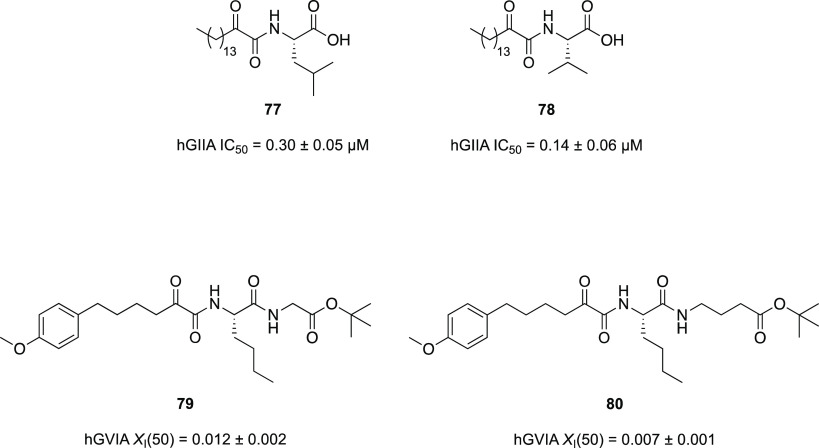
Structures and biological activities of GIIA sPLA_2_ (**77**–**78**) and GVIA iPLA_2_ (**79**–**80**) inhibitors.^159,162,163^

In the same year, Vasilakaki et al.^162^ tried to improve the activity of the previously reported compound **77** (Figure 25)^159^ that showed activity in the low micromolar range against hGIIA and hGVA sPLA_2_s. With the aid of molecular docking calculations and bearing in mind the SARs demonstrated in the previous work, researchers developed a new series of 2-oxoamides based on nonpolar α-amino acids having (*S*)-configuration.^162^ Among all the new compounds, only **78** (Figure 25) showed improvements in potency compared to **77** (IC_50_ of 0.14 and 0.30 μM, respectively) against human GIIA sPLA_2_ and was selective against this isozyme without affecting other human and mouse sPLA_2_s.

Replacing the long aliphatic chain by a shorter one carrying an aromatic system (structures not shown) was detrimental for the activity. Computational analysis revealed that the long aliphatic chain maintains the oxoamide moiety close to the fundamental residues of the catalytic site. Shorter chains allow the moiety to move impacting the activity against sPLA_2_s.

Smyrniotou et al. investigated the 2-oxoamide moiety to develop inhibitors against GVIA iPLA_2_.^163^ From the studies performed so far, they noticed that some ester analogues of potent GIVA cPLA_2_ inhibitors showed some inhibition against GVIA iPLA_2_. In addition, 2-oxoamide-based compounds featuring dipeptides or ether dipeptides showed a slight preference for the isozyme they wanted to inhibit. Thus, they designed compounds based on 2-oxoamide functionality accompanied by a small peptide unit.^163^ This peptide unit was based on nonpolar amino acids, which create favorable interactions with the active site of the GVIA iPLA_2_. Plus, they attached an aromatic moiety (phenyl, unsubstituted or bearing a *p*-methoxy group, or naphthalene ring) at four carbon atoms of distance from the activated carbonyl. This distance demonstrated to be optimal by previous studies on polyfluoroketone derivatives. From the first series of compounds analyzed, **79** (Figure 25) was the only one showing an inhibition against the desired isozyme superior at 95% with a *X*_I_(50) of 0.012.^163^ Moreover, **79** weakly inhibited the other two forms GIVA cPLA_2_ and GV sPLA_2_. Further modifications of **79** were then explored. Replacement of the *tert*-butyl ester moiety led to decreased inhibitory activity. Analogously, modifying the length of the peptide unit killed the activity. Then, the researchers tried to modify the dipeptide unit, first replacing Nle with other amino acids containing small aliphatic chains, without success. Only introducing a Leu residue produced interesting activity but still half as potent as **79**. Modification of Gly portion, maintaining Nle, led to compound **80** (Figure 25) having a dipeptide structure Nle-GABA-OBut. Modification of the ester moiety did not lead to better inhibitory activity. **80** showed 13 times more potent inhibition of GVIA iPLA_2_ than GIVA cPLA_2_, and its inhibition [*X*_I_(50) = 0.007] is comparable with that of two commercially available inhibitors of GVIA iPLA_2_, FKGK11 [*X*_I_ (50) 0.0014], and AACOCF_3_ [*X*_I_ (50) 0.028].^163^

#### 3.1.2 Gastric and Pancreatic Lipases’ Inhibitors

Lipases are ubiquitously expressed enzymes found in animals, plants, fungi, and bacteria. Human lipases are secreted by exocrine glands of pancreas and catalyze hydrolysis of the ester bonds of triglycerides. Pancreatic and gastric lipases play a crucial role for fat digestion in humans and higher animals. Hydrolysis of dietary triglycerides to monoacylglycerols and free fatty acids catalyzed by these enzymes is mandatory for fat absorption by the enterocytes.^165^ Because of their importance to fat digestion, lipases have been targeted for the development of inhibitors to fight obesity. The catalytic active site consists of a triad (Ser–His–Asp) homologous to that proposed for serine proteases and an oxyanion hole, which stabilizes the transition state.

Thus, the glyoxylamide moiety may be introduced as an electrophilic group to mimic the scissile ester group of the natural lipase substrate.

**Figure 26:**
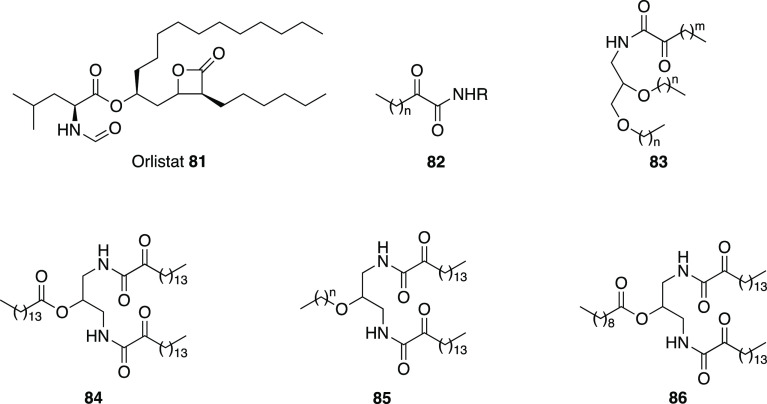
Structures of orlistat **81** and lipase inhibitors **82**–**86**.^165^

The only approved drug for long-term treatment of obesity to date is orlistat **81** (Figure 26), a pancreatic lipase inhibitor. It is a saturated derivative of lipstatin, and its mechanism of action consists of binding covalently to Ser152 of the active site of the enzyme by its β-lactone ring. Even if it has been reported to have tolerable drawbacks, its long-term use has been associated with severe adverse effects (hepatotoxicity, gall stones, and acute pancreatitis, among others). For this reason, research continues in order to achieve improved molecules.^164^

In 2003, Kokotos et al. published a review reporting the results achieved by his group involving the investigation of 2-oxoamide-based inhibitors of these enzymes.^166−168^ Lipase inhibitors’ structure should contain two components: an electrophilic moiety being able to react with the serine belonging to the active site, and a lipophilic segment mimicking the natural substrate, differently decorated to improve interaction and orientation into the binding pockets of the enzyme. The glyoxylamide moiety was introduced as an electrophilic group to mimic the scissile ester group of the natural lipase substrate. Along with a series of *N*-alkyl-2-oxoamides **82**, derivatives **83** and bis-2-oxo amide triacylglycerol analogues **84** and **85** were developed (Figure 26). When evaluated for their capability to inhibit pancreatic and gastric lipases, these compounds showed a weak inhibition against porcine pancreatic lipases (PPL), with no significant differences among the explored substitutions.^166−168^ Results were expressed as inhibitor molar fraction value (α_50_) corresponding to the inhibitor molar fraction present in 1,2-dicaprin monolayers that causes a 50% decrease in the enzymatic activity. However, results for human gastric lipase (HGL) showed differences correlated to the chirality of the molecule: (*R*)-enantiomers were 2-fold better inhibitors of the corresponding molecules having (*S*)-configuration. HGL showed a preference for bis-2-oxoamides, particularly for bis-2-oxoamide triacylglycerol analogues **84** and demonstrated 4–5-fold more potency than the corresponding ethers **85**. This finding could suggest an importance for the ester oxygen in the interaction with the enzyme. Compound **86** (Figure 26) was the most potent against this enzyme (α_50_ = 0.020), even though it was a weak inhibitor compared to **81**, which shows an α_50_ value of 0.0025.^166−168^

**Figure 27:**
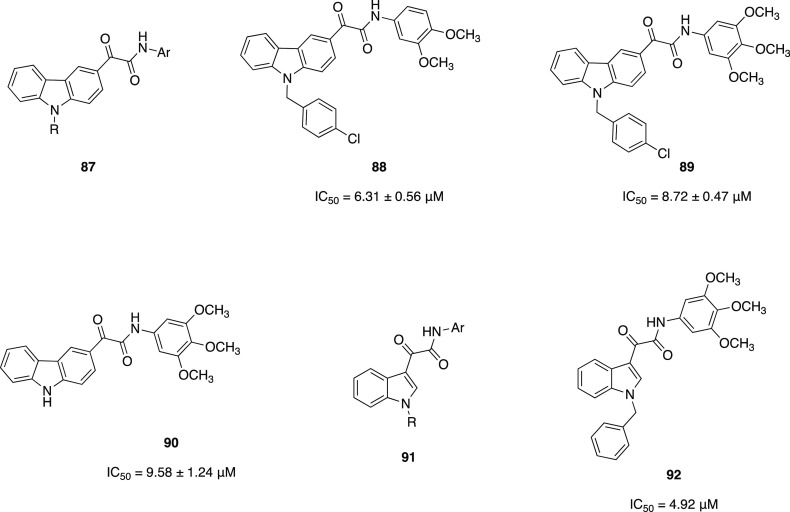
Structures and biological activities of 2-(carbazol-3-yl)-2-oxoacetamides **87**–**92**.^169,170^

In 2017, Sridhar et al. also investigated the 2-oxoamide moiety as an ester mimicking group in the field of pancreatic lipase (PL) inhibitors.^169^ In particular, this moiety was combined with a carbazole scaffold, which gained attention in recent years for the wide range of biological activity, including PL inhibition.^169^ A series of carbazolyl oxoacetamides (**87**, Figure 27) was developed with various substituent attached to the carbazolyl nitrogen and the aromatic substituent at the 2-oxoamide moiety.

When compounds were evaluated for their inhibitory activity toward porcine PL (**81** was taken as reference compound), the general trend observed was that an electron withdrawing substituent on the carbazolyl nitrogen, as well as an electron donating group on the oxoamide nitrogen, improves the activity. The three most potent compounds were **88**, **89**, and **90** (Figure 27) with IC_50_ values of 6.31 ± 0.56 μM, 8.72 ± 0.47 μM, and 9.58 ± 1.24 μM, respectively, though still far from that of **81** (Figure 26, IC_50_ 0.99 ± 0.11 μM). Compounds **88**, **89**, and **90** were evaluated to investigate the nature of inhibition. All three compounds were shown to inhibit PL competitively, as well as **81**, confirming their bond to the enzyme active site.^169^ In addition, MD studies validated the crucial role of the α-ketoamide moiety to react in a covalent manner with Ser 152 of the active site, similarly to **81**.^169^ The superimposition of the binding mode of **88** on that of **81** showed that the reactive carbonyl groups of both compounds were overlapping each other with a minor deviation (<1 Å), proving a potential covalent interaction of **88**, similarly to **81**. Nonetheless, this *in silico* study evidenced a steric hindrance exerted by the carbazole ring, which led to an increased interaction distance between the reactive carbonyl group of the 2-ketoamide and Ser152. The same research group replaced the carbazole core with an indole nucleus with the aim to decrease this steric hindrance and potentially enhance the PL inhibitory activity.^170^ A series of indole glyoxylamides **91** (Figure 27) was developed and tested for their ability to inhibit porcine PL, using **81** as a reference. The most active compound of this series, **92** (IC_50_ 4.92 μM, Figure 27), when subjected to an enzymatic kinetic assay against the substrate, showed a competitive inhibition like **81** and the previous class, confirming its bonding to the active site of PL.^170^ Furthermore, the interaction distance between the reactive carbonyl group and Ser152 was shown to play a crucial role in the PL inhibition. Indeed, this distance was lesser for indole **92** (3.84 Å) with respect to carbazole **88** (4.45 Å), while carbonyl group of **81**’s β-lactone was at 3.3 Å from Ser152. These results confirmed that the replacement of carbazole with an indole nucleus diminished the interaction distance, resulting in potentiated PL inhibitors (**88**: 6.31 μM; **92**: 4.92 μM).

#### 3.1.3 Hepatitis C NS3/4A Protease Inhibitors

Hepatitis C is an infection caused by the hepatitis C virus (HCV), which causes acute and chronic necroinflammatory liver diseases. HCV infections have reached pandemic proportions with 71 million HCV-infected patients globally, 1.75 million individuals newly infected in 2015, and an estimated 390,000 people have died from HCV infection.^171^ A member of the Flaviviridae family, HCV is an uncapped, linear, single-stranded RNA (ssRNA) molecule with positive polarity that serves as a template for both translation and replication. The HCV genome encodes a polyprotein of structural and nonstructural (NS) proteins.^172^ The virally encoded HCV NS3/4A chymotrypsin-like serine protease is activated by the noncovalent association of NS3 with its cofactor NS4A. It contains a canonical Asp-His-Ser catalytic triad, it is responsible for the processing of the HCV polyprotein, and it has been recognized as a promising target to design new anti HCV drugs due to its pivotal role in viral replication.^173^

In this context, the α-ketoamide moiety may be introduced as electrophilic group to the scissile amide bond of the natural substrate.

Before 2011, the standard of care for HCV consisted of a weekly injection of the pegylated interferon-α (PEG-IFN-α) combined with daily oral doses of the broad-spectrum antiviral ribavirin (RBV). However, this combination therapy possesses several limitations that prompted researchers to look for new anti-HCV drugs with improved efficacy and tolerability.

**Figure 28:**
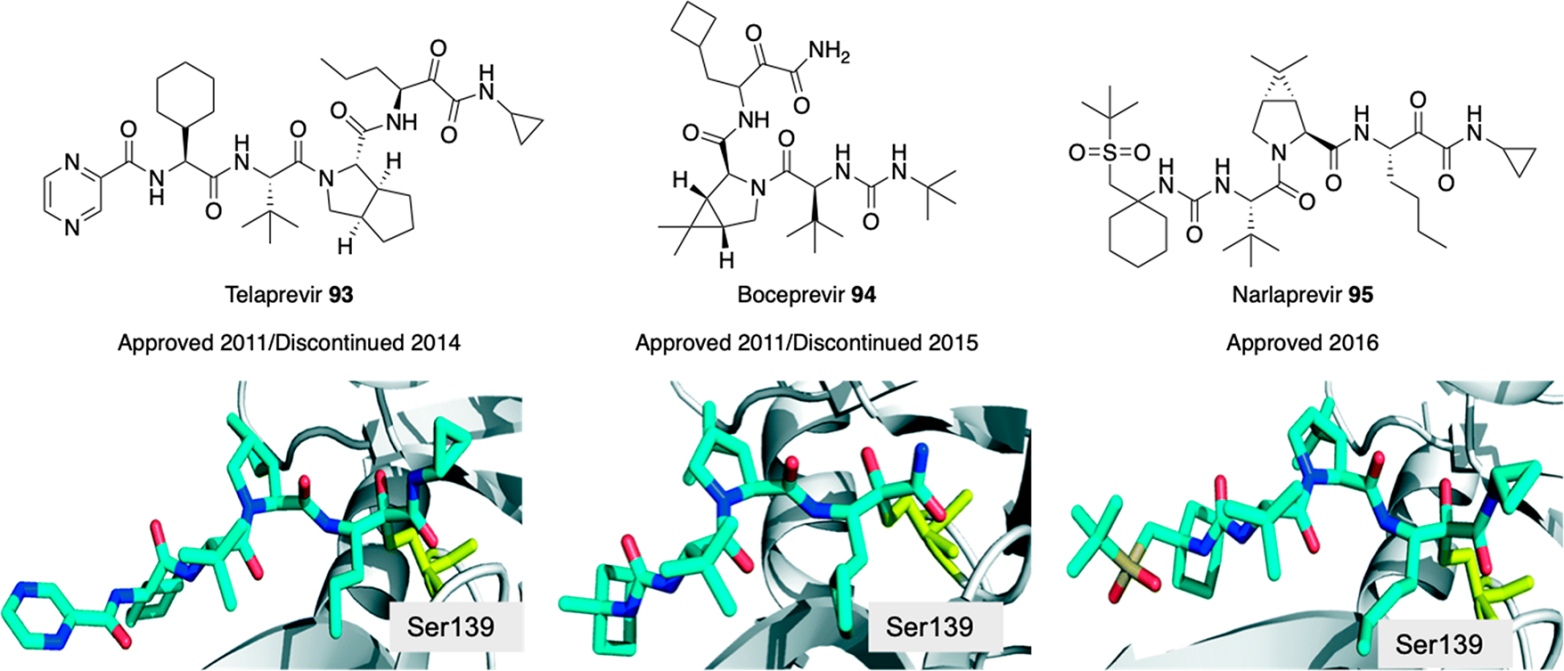
Structures of reversible covalent protease inhibitors **93**–**95** and their crystal structures with the NS3/4A protease complex.^177^**93** (PDB ID 3SV6), **94** (PDB ID 3LOX), and **95** (PDB ID 3LON) binding to Ser139. Inhibitors are shown as cyan sticks and Ser139 is shown as yellow sticks. Reproduced from ref (177), under Attribution 3.0 Unreported, CC BY-NC 3.0.

In 2011, the first-generation HCV NS3/4A reversible covalent protease inhibitors telaprevir (TVR, **93**, Figure 28)^174^ and boceprevir (BCV, **94**, Figure 28)^175^ were approved to be used in combination with PEG-IFN-α and RBV for treatment of HCV infection in patients with genotype 1 of chronic hepatitis C (CHC). The cure rate increased to 75% with **93** and to 70% with **94**. In addition, the treatment was reduced from 48 weeks to 24–28 weeks. More recently, Yamada et al. demonstrated that **93** in combination with PEG-IFNα-2a/RBV provide a sustained viral response (SVR) in both treatment naïve and previously treated patients. Moreover, **93**-based therapy may offer a favorable treatment for patients who are infected with treatment-resistant variants.^176^

Epimerization at the chiral center adjacent to the α-ketoamide of **93** leads to formation of its main metabolite, the *R*-diastereoisomer, which showed a 30-fold reduction of activity against HCV protease. In this context, with the aim to modulate such epimerization, without losing the activity, the chiral proton of **93** was replaced with deuterium (d). Deuterium substitution resulted in a more stable compound than **93**, under basic conditions and in plasma, without altering in vitro antiviral properties. In addition, oral administration in rats resulted in a 13% increase of AUC for d-**93**.^178^

Narlaprevir **95** (Figure 28) is a potent second-generation reversible covalent inhibitor of HCV NS3 protease and was approved in 2016 for the treatment of genotype 1 HCV. In clinical trials, it caused a quick and steady reduction in viral RNA levels in both relapsed and naïve patients when used in combination with PEG-IFN-α. Additionally, it also proved to be active against HCV mutation resistant to other treatments such as **94** and **95**.^179^ An important feature common to this class of molecules is the presence of α-ketoamide warhead that is responsible for the formation of reversible covalent bond with the catalytic residue in the active site (Figure 28).

#### 3.1.4 Dengue Virus Proteases

Dengue virus (DenV) belongs to the family Flaviviridae, consists of a positive-single stranded RNA genome, and produces a severely neglected tropical disease, Dengue fever.^180,181^ During viral replication, the DenV genome encodes for a viral precursor single-polyprotein, which must be cleaved into functional proteins by host proteases and viral serine protease, specifically a complex of the NS3 protein with its cofactor NS2B. As this cleavage is essential for the viral life cycle, NS2B/NS3 protease, a serine endoprotease that belongs to the chymotrypsin family with the catalytic triad His51-Asp75-Ser135, represents an attractive target for the development of DenV therapeutics.^182^

**Figure 29:**
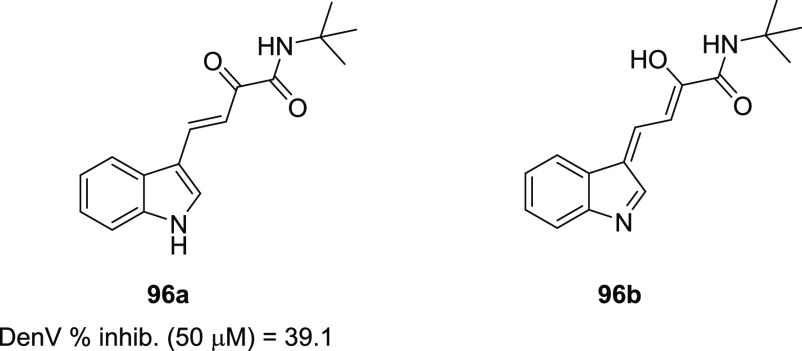
Structure and biological activity of derivative **96a** and a possible tautomer **96b**.

In this context, tetrameric or larger peptide derivatives combined with aldehydes as an electrophilic group were developed; unfortunately, these compounds did not demonstrate the desired drug-like properties.^183,184^ Klein and colleagues exploited the replacement of the aldehyde group with the α-ketoamide moiety with the aim to develop viral proteases inhibitors with improved drug-likeness.^185^ Several β,γ-unsaturated α-ketoamides were synthesized, and SARs clearly evidenced the crucial role of the α-ketoamide function for the biological activity, α-hydroxy and α-epoxy derivatives being far less effective in the virus inhibition. Although the majority of compounds exhibited only moderate DenV proteases inhibition in the enzymatic assay, the most interesting derivative **96a** (Figure 29) showed the ability to inhibit DenV replication in a cell-culture assay in a concentration-dependent manner that resulted in a more than 1000-fold reduction of virus load at noncytotoxic concentrations. It should be speculated that the weak activity of this compound might be correlated to the presence of the double bond at the α,β-position with respect to the ketocarbonyl that makes possible the existence of inactive tautomers, such as **96b** (Figure 29).

### 3.2 Cysteine Proteases

#### 3.2.1 3C or 3C-like Protease Inhibitors

Positive-sense RNA viruses have their genetic material directly translated into one or more polyproteins that are cleaved into mature or intermediate viral proteins by viral proteases. The picornavirus-like supercluster includes some of these positive-sense RNA viruses, including viruses belonging to the *Picornaviridae, Caliciviridae*, and *Coronaviridae* families. Examples of these viruses are the Norwalk virus (NV) and feline calicivirus (FCV) (*Caliciviridae* family); enterovirus 71 (EV71), poliovirus (PV), foot-and-mouth disease virus (FMDV) and hepatitis A virus (HAV) (*Picornaviridae* family); and human coronavirus 229E, transmissible gastroenteritis virus (TGEV), bovine coronavirus (BCV), feline infectious peritonitis virus (FIPV), and severe acute respiratory syndrome coronavirus (SARS-CoV) (*Coronaviridae* family).^186^ A common feature among viruses of picornavirus-like supercluster is the possession of a viral 3C or 3C-like protease (3Cpro or 3CLpro, respectively) that is responsible for the aforementioned cleavage of viral polyproteins into mature or intermediate viral proteins. These two enzymes are both cysteine proteases and share several common features, including a Cys residue as an active site nucleophile in the catalytic triad (or dyad), composed of Cys, His, and Glu (or Asp) residues, and the substrate binding pockets with a preference for a Glu or Gln residue at the P1 position on the substrate.^186^ Introduction of an electrophilic group mimicking the scissile amide bond of the natural substrate, such as the 2-oxoamide moiety, may permit rational design of novel inhibitors of cysteine proteases.

Noroviruses, belonging to the Norovirus genus of the *Caliciviridae* family, are highly contagious human pathogens, commonly involved in foodborne and waterborne acute gastroenteritis. Norovirus 3CLpro is a cysteine endoprotease with a catalytic triad composed of Cys-His-Glu residues. X-ray crystal structures of the enzyme alone or covalently bound to inhibitors, such as Michael acceptor and peptidyl aldehydes, have been reported.^187^ In an attempt to develop molecules with favorable ADMET properties and suitable features for oral bioavailability, Mandadapu et al. developed a series of peptidyl α-ketoamides and α-ketoheterocycles. These molecules showed comparable antiviral activity against norovirus 3CLpro in vitro compared to previously reported aldehyde inhibitors, and a 10-fold increment in potency in a cell-based replicon system (**97**–**99**, Table 8).^187^

**Table 8:**
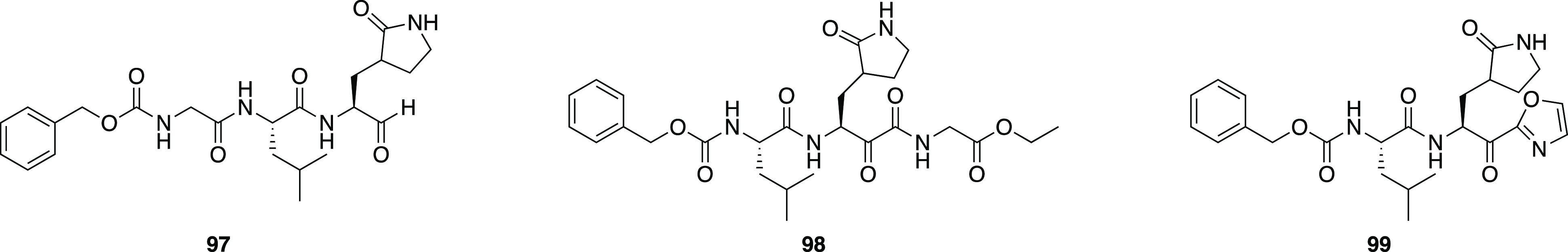
Inhibitory Activity of Peptidyl Derivatives against Norovirus 3CLpro in Vitro (IC_50_) and in Cell-Based Replicon System (ED_50_)^a^

cpd	IC_50_ [μM]	ED_50_ [μM]
**97**	1.45	7.8
**98**	2.1	0.8
**99**	2.3	0.9

a Data taken from Mandadapu et al.^187^

**Figure 30:**
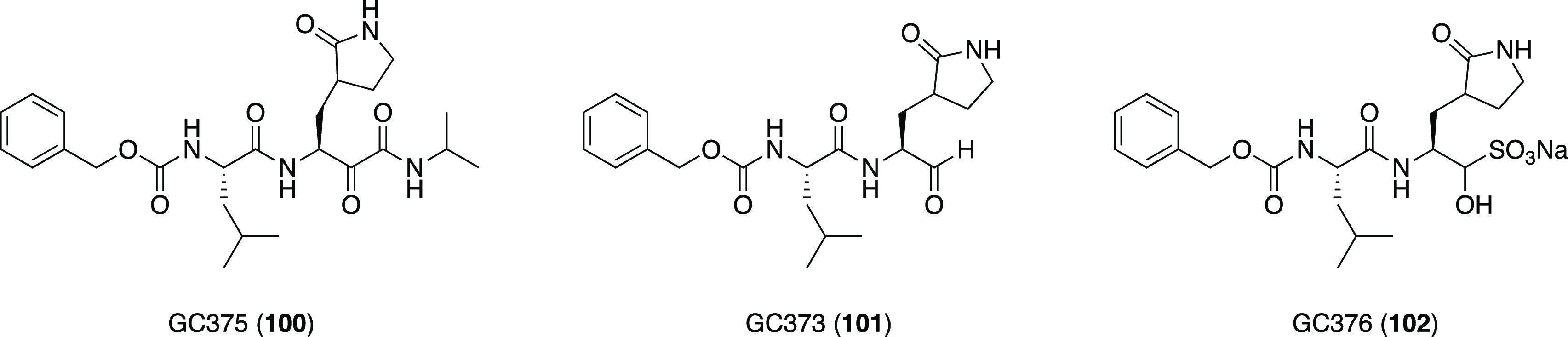
Structures of dipeptidyl derivatives **100**-**102** investigated by Kim et al.^186^

Among the series developed by Mandadapu et al., **100** (GC375, Figure 30) was chosen along with other two dipeptidyls **101** and **102** (GC373 and GC376, Figure 30), bearing a Gln mimicking structure in a position that corresponds to the P1 position and a Leu in the P2 position (in the nomenclature of Schechter and Berger^188^), to be tested as inhibitors against a wide panel of viruses from picornavirus-like supercluster.^186^

In the enzyme- and/or cell-based studies set up to evaluate the capability of these derivatives to inhibit viral replication or viral protease activity, the α-ketoamide **100** showed IC_50_ values in the low-micromolar/high-nanomolar range against coronaviruses and picornaviruses, comparable to **101** and **102**. The weaker activity of the α-ketoamide warhead against caliciviruses shown by this study has been ascribed to its excessive bulkiness to fit in the active site of the target protein.^186^

A series of subsequent studies described a better activity against picornaviruses and coronaviruses, and these studies are summarized below.

Prior et al. developed a set of tripeptidyl transition state inhibitors featuring an aldehyde warhead of glutamine surrogate at P1, leucine at P2, and arylalanine at P3. In this series, different warheads were also evaluated in order to prevent oxidative degradation and ameliorate absorption and *in vivo* PK, compounds **103**–**106** (Table 9).^189^

**Table 9:**
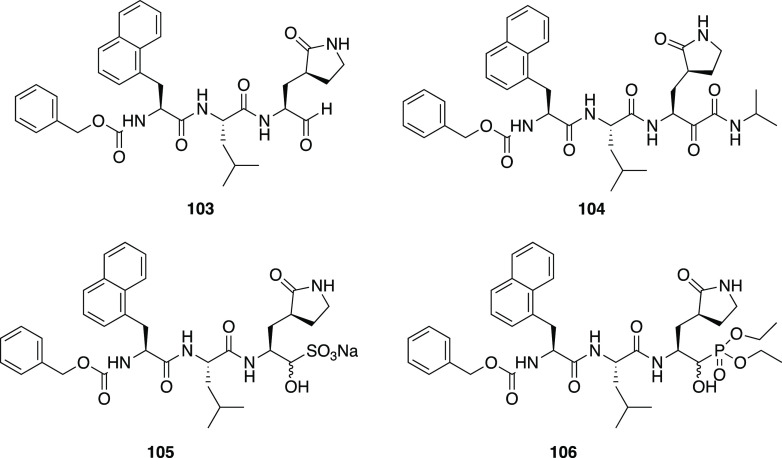
IC_50_ and EC_50_ Values of Compounds **103**–**106** from NV 3CLpro Fluorescence Resonance Energy Transfer (FRET)-Based Assay and NV Replicon Cells, Respectively^a^

cpd	IC_50_ [μM]	EC_50_ [μM]
**103**	0.14 ± 0.2	0.04 ± 0.02
**104**	2.6 ± 1.5	4.2 ± 2.4
**105**	0.24 ± 0.1	0.04 ± 0.03
**106**	35.5 ± 5.7	0.1 ± 0.3

a Data taken from Prior et al.^189^

The α-ketoamide **104** performed poorly compared to the aldehyde counterpart **103**, as reported in Table 9. Bisulfite adduct **105** is a precursor and pro-drug of **103** through equilibrium in aqueous solution. α-Hydroxy phosphonate **106** showed potent activity in NV replicon cells. When evaluated against a panel of viruses belonging to picornavirus and coronavirus families, the α-ketoamide **104** showed improved antiviral activity, comparable to the aldehyde warhead **103**. Furthermore, **104** was demonstrated to be less toxic in a cell-based assay using a NV replicon cell system (Table 10).^189^

**Table 10:** (Top) IC_50_ Values Compounds **103** and **104** against Various 3Cpro and 3CLpro Using FRET-Based Enzyme Assays and (Bottom) EC_50_ Values of Compounds **103** and **104** against Various Viruses in the Cell-Based Assays and CC_50_ (Concentration That Causes 50% Cell Death) Values^a^

cpd	NV [μM]	MD145 [μM]	HRV [μM]	SARS-CoV [μM]
**103**	0.14 ± 0.2	0.51 ± 0.2	0.15 ± 0.05	0.23 ± 0.1
**104**	2.6 ± 1.5	3.1 ± 1.6	0.12 ± 0.2	0.61 ± 0.2

cpd	NV [μM]	MNV-1 [μM]	HRV18 [μM]	coronavirus 229E [μM]	CC_50_ [μM]
**103**	0.04 ± 0.02	0.6 ± 0.2	0.015 ± 0.03	0.2 ± 0.1	87 ± 6.3
**104**	4.2 ± 2.4	0.03 ± 0.04	0.03 ± 0.04	0.5 ± 0.2	>100

a Data are taken from Prior et al.^189^

**Figure 31:**
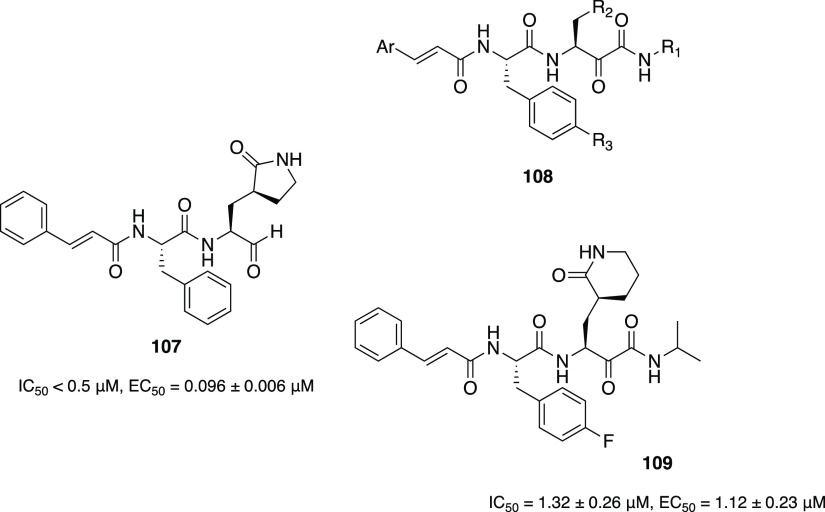
Structures of aldehydes and glyoxamide derivatives as EV71 3Cpro inhibitors, and IC_50_ and EC_50_ values of compounds **107** and **109** from EV71 3Cpro FRET-based assay and EV71 replicon cells, respectively.^14,192^

The enterovirus 71 (EV71) is one of the main causes of hand, foot, and mouth disease (HFMD), and it belongs to the family of picornaviruses. It is a mild, contagious viral illness that occurs in all areas of the world and usually affects infants and children younger than 5 years old, although it can occasionally occur in older children and adults. Common symptoms are fever, mouth sores, and a skin rash on the hands and feet, and no specific treatments are currently available.^190^ As for other viruses of this family, the 3C proteases’ (3Cpro) critical role in EV71 infection makes it an attractive target for drug discovery.^191^ In order to inhibit the EV71 3Cpro, Zeng et al. investigated a series of derivatives bearing the α-ketoamide moiety, whose functionalization allowed SAR investigation of the P1′ site interacting with S1′ pocket of 3C protease, along with modifications to P1 and P3. On the basis of previously reported aldehyde inhibitors, showing inhibitory activity in the nanomolar range both in vitro and in cell-based assays (**107**, IC_50_ < 0.5 μM, EC_50_ 0.096 ± 0.006 μM, Figure 28),^192^ a library of α-ketoamides **108** (Figure 31) was developed and tested in vitro against EV71 3Cpro.^14^

**Figure 32:**
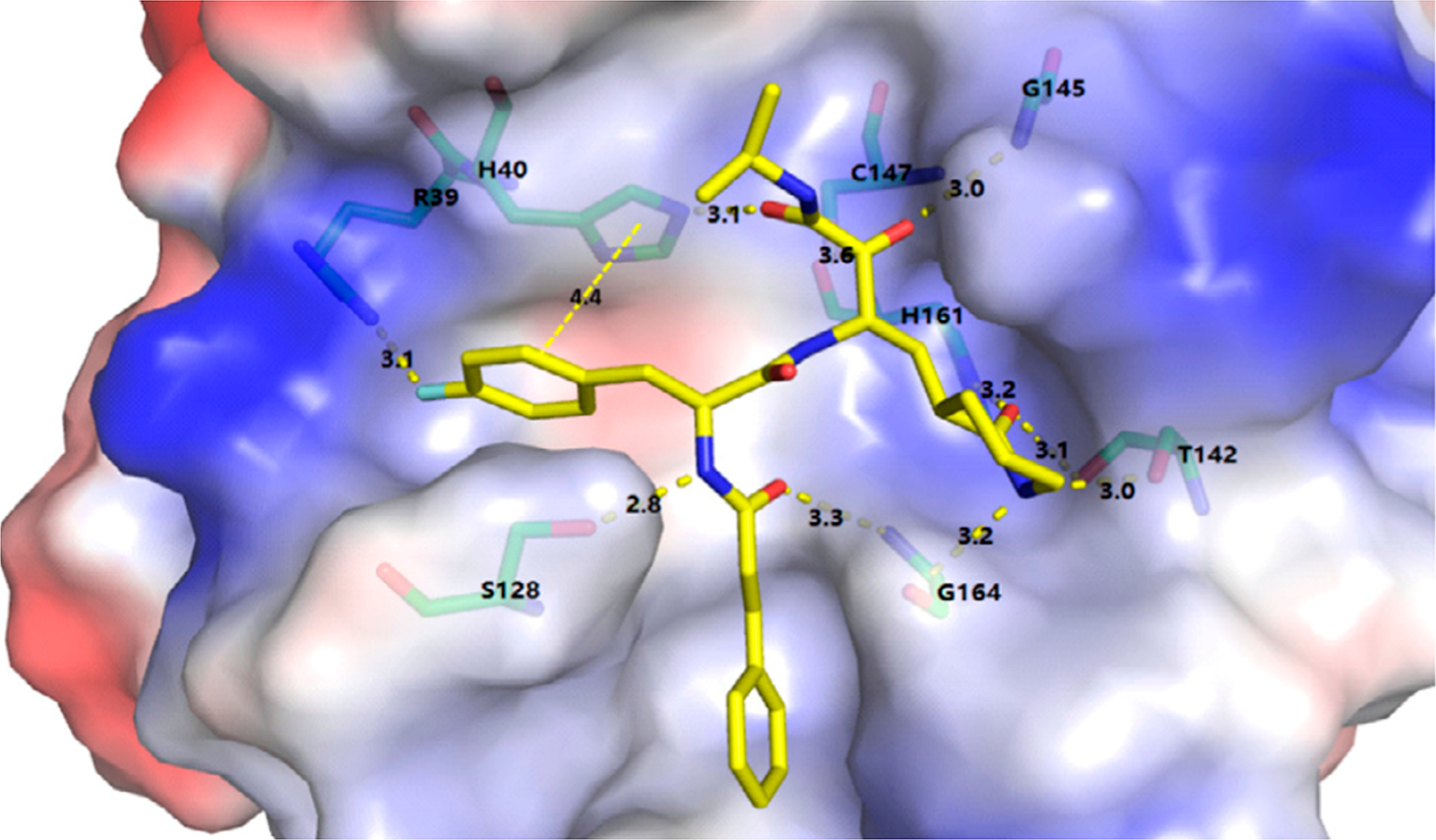
Docking model of **109** bound to EV71 3Cpro.^14^ Reproduced with permission from ref (14). Copyright 2016 Elsevier.

In general, all the α-ketoamides **108** were less potent inhibitors with respect to the previously reported aldehyde derivatives. The replacement of the (*S*)-γ-lactam ring by (*S*)-δ-lactam one at the P1 position (R_2_) improved the potency of inhibitors against EV71 3Cpro. In addition, the presence of a short and small branched terminal chain at the R_1_ position resulted in more potent compounds. Furthermore, the presence of a *p*-fluorobenzyl group instead of a benzyl one at P2 notably increased the inhibitor potency by 2–3 fold (**109**, IC_50_ 1.32 ± 0.26 μM, EC_50_ 1.12 ± 0.23 μM, Figure 31). Replacement of the styrene moiety at P3 with a carbobenzoxy or *t*-butyloxycarbonyl one produced compounds with comparable potency, suggesting the variation at P3 has less effect on inhibitor activity with respect to P1, P2, and P1′. All the α-ketoamides exerted low toxicity in the *in vitro* cytotoxicity assay (CC_50_ > 100 μM). Molecular docking studies on **109** (Figure 32) elucidated the role of the α-ketoamide moiety in forming favorable hydrogen bonds between keto-carbonyl and Gly145 and amide carbonyl and His40 in the active site, enhancing the electrophilicity, thus the reactivity, of the moiety itself toward nucleophilic attack from the catalytic Cys residue. All these results highlighted the α-ketoamide as a good choice in the field of EV71 3Cpro inhibitors.^14^

**Figure 33:**
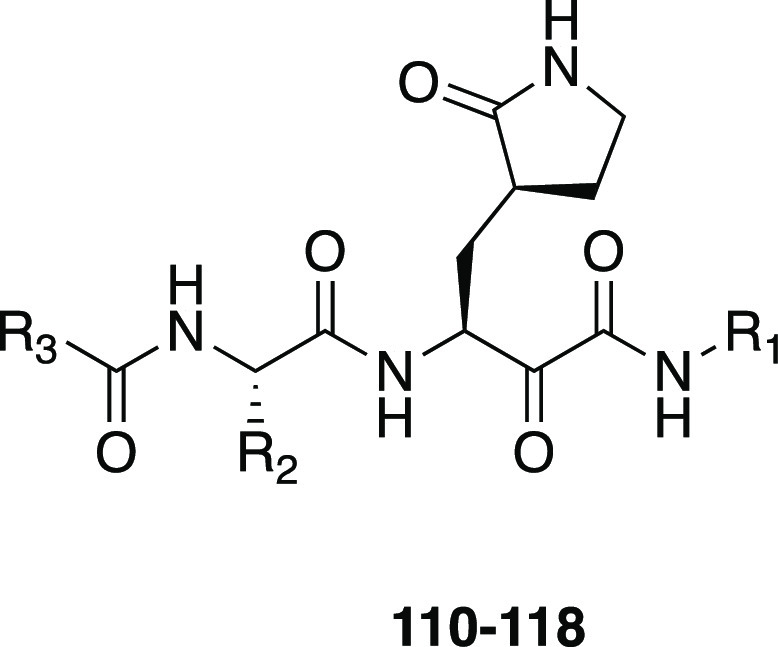
Structures of α-ketoamides **110**–**118** developed by Zhang et al.^193^

Recently, Zhang et al. investigated the antiviral effects of the 2-oxoamide moiety on different viral proteases belonging to coronaviruses and enteroviruses. Analyzing crystal structures of several viral proteases, researchers put at the P1 position a five-membered ring (γ-lactam) derivative of glutamine in their α-ketoamides, then focusing on the substitution at the P1′, P2, and P3 positions (R_1_, R_2_, and R_3_, respectively, **110**–**118**, Figure 33).^193^

Compounds were tested against four different viral proteases from enterovirus A71, coxsackievirus B3, HCoV NL63, and SARS-CoV, outlying the importance of benzyl and cinnamoyl moieties at the P1′ and P3 position, respectively (**110**–**115**, Figure 33). Derivatives **110**–**115** possessed the best overall activities against the viral proteases (Table 11), so the tests proceeded against viral replicons and against SARS-CoV, MERS-CoV, and a wide set of enteroviruses in cell culture-based assays.^193^

**Table 11:**
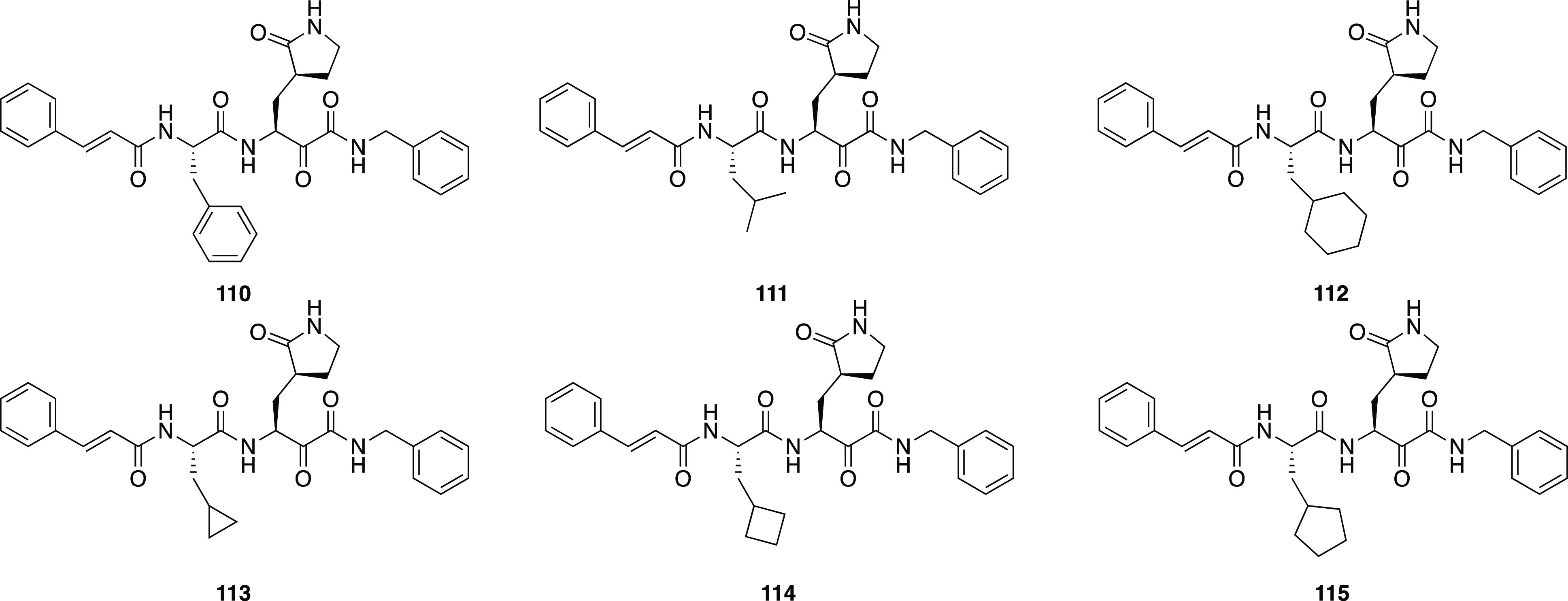
Antiviral Activities against 3CLpro and Mpro (Main Protease) of **110**–**115**^a^

cpd	EV-A71 [μM]	CVB3 [μM]	SARS-CoV [μM]	HCoV-NL63 [μM]
**110**	1.22 ± 0.12	6.56 ± 3.10	1.95 ± 0.24	>50
**111**	13.80 ± 4.17	3.82 ± 1.21	0.33 ± 0.04	1.08 ± 0.09
**112**	1.69 ± 0.47	0.95 ± 0.15	0.71 ± 0.36	12.27 ± 3.56
**113**	18.47 ± 4.25	4.25 ± 1.40	0.24 ± 0.08	1.37 ± 0.35
**114**	10.81 ± 2.62	4.78 ± 1.13	1.44 ± 0.40	3.43 ± 2.45
**115**	4.73 ± 0.94	1.93 ± 0.43	1.27 ± 0.34	5.41 ± 2.31

a Data are taken from Zhang et al.^193^

**Figure 34:**
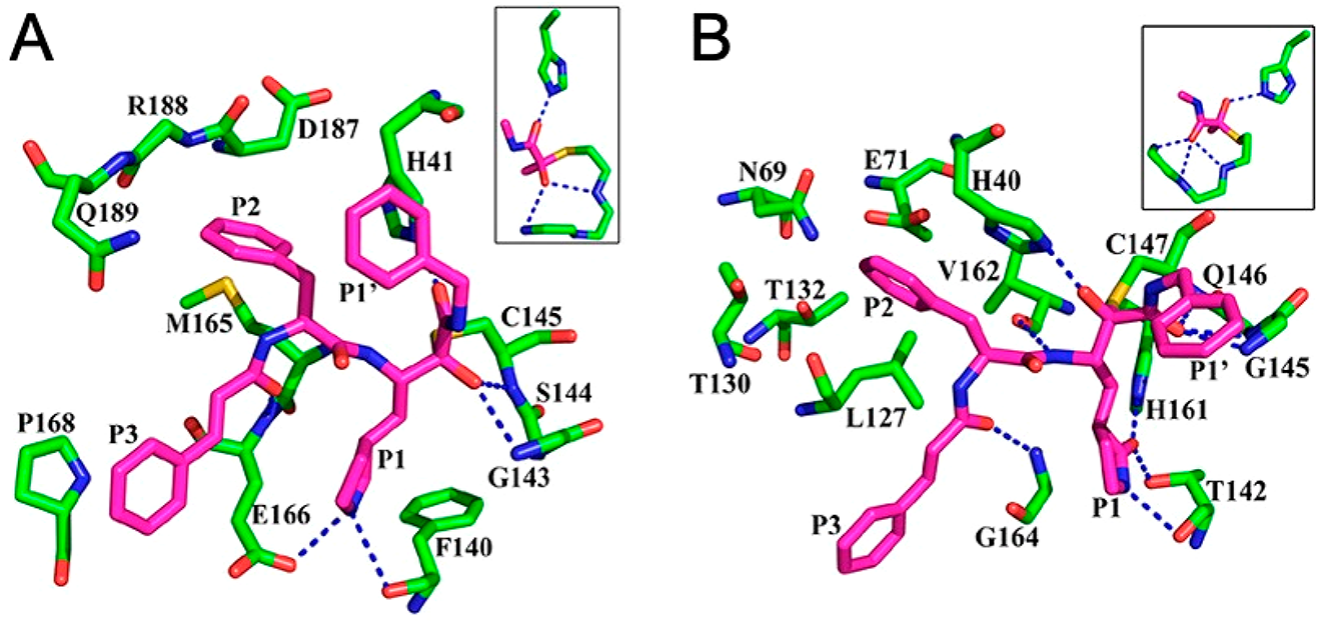
Detailed interactions of peptidomimetic α-ketoamide **110** (pink carbon atoms) with target proteases (green carbon atoms). Hydrogen bonds are depicted as blue dashed lines. The inset at the top of the images shows the configuration of the thiohemiketal formed by the nucleophilic attack of the catalytic Cys residue onto the α-keto group. (A) Binding of **110** to SARS-CoV M^pro^. The thiohemiketal is in the *R* configuration, with its oxygen accepting two hydrogen bonds from the oxyanion-hole amides of Gly143 and Cys145. The amide oxygen accepts an H-bond from His41. The side chains of Ser144 and Arg188 have been omitted for clarity. (B) Binding of **110** to the CVB3 3C^pro^. The stereochemistry of the thiohemiketal is *S*, as the group accepts a hydrogen bond from His41, whereas the amide keto group accepts three H-bonds from the oxyanion hole (residues 145–147). The side chain of Gln146 has been omitted for clarity. Reproduced from ref (193) that is made available via the ACS COVID-19 subset for unrestricted RESEARCH reuse and analyses in any form or by any means with acknowledgment of the original source. These permissions are granted for the duration of the World Health Organization (WHO) declaration of COVID-19 as a global pandemic.

Data obtained in cell-based assays confirmed the overall low-micromolar activity of these compounds, with the excellent activity values of **111** and **112** against MERS-CoV in Huh7 cells (EC_50_ 4.8 nM and 0.4 nM, respectively). Particularly, **112** showed the best activity in all the cell lines (except for HCoV-229E against which **111** performed better) along with weak toxicity, so that it has been chosen for future development. Preliminary pharmacokinetic tests did not highlight a toxicity problem in mice. Most importantly, in accordance with the aim of the present perspective work, Zhang et al. found by means of crystallographic analyses that α-ketoamide warheads are sterically more adaptable than other warheads like Michael acceptors and aldehydes. This is caused by the presence of two H-bond acceptor sites, namely, the α-keto oxygen and the amide oxygen, while the other moieties feature only one such acceptor. In the various complexes, once the active-site cysteine residue carries out the nucleophilic attack onto the α-keto carbon, the hydroxy group (or oxyanion) of the thiohemiketal becomes able to accept one or two hydrogen bonds from the main-chain amides of the oxyanion hole. Furthermore, the catalytic His residue can form a hydrogen bond with the amide oxygen of the inhibitor. The two interactions here described can also be switched, having an interaction between the thiohemiketal and the catalytic His residue, and the amide oxygen with the main-chain amides of the oxyanion hole. The interaction will affect the stereochemistry at the thiohemiketal C atom (interactions of compound **110** are depicted in Figure 34 as an example).^193^

**Figure 35:**
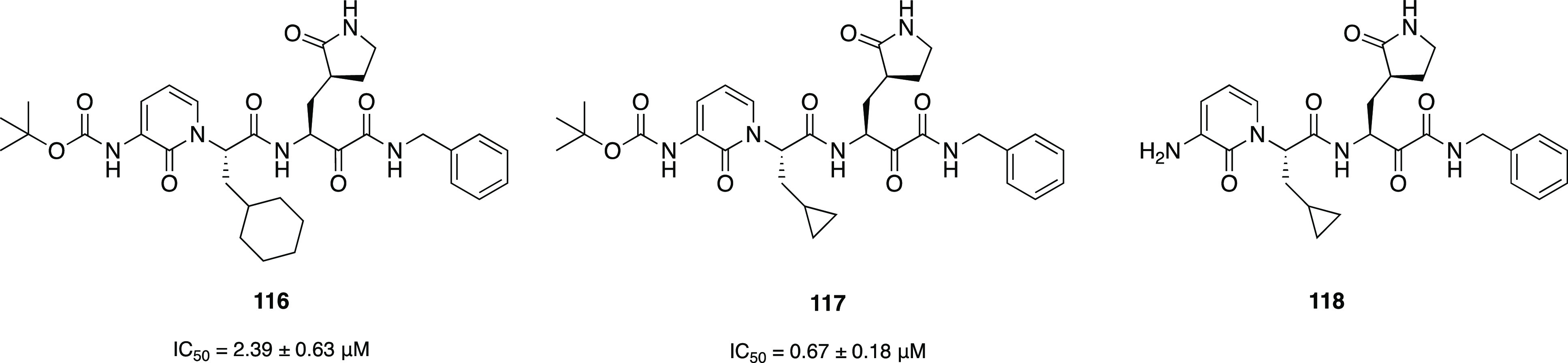
Structures and biological activities of derivatives **116**–**118** developed by Zhang et al.^194^ against the novel SARS-CoV-2.

Compound **112** was also investigated against the novel SARS-CoV-2 responsible for the recent global pandemic, since the molecule showed low micromolar activity against SARS-CoV and the novel virus shares about 82% of the RNA genome with the previous pathogen.^194^ Zhang et al. showed that the α-ketoamide **112** inhibits the main protease (Mpro) of SARS-CoV-2 with an IC_50_ value of 0.18 ± 0.02 μM. In order to improve the pharmacokinetic profile of the molecule, a pyridone ring between P3 and P2 was introduced, trying to prevent the cleavage protease-mediated of the amide bond. Then, the cinnamoyl moiety was replaced by the less hydrophobic Boc group in order to improve plasma solubility and to reduce the binding to plasma proteins, obtaining compound **116** (Figure 35).^194^

These modifications led to an increased plasma half-life, improved in vitro kinetic plasma solubility, and enhanced thermodynamic solubility. On the other hand, the inhibitory activity against SARS-CoV-2 Mpro decreased to 2.39 ± 0.63 μM, because the molecule retained the cyclohexyl moiety at the P2 position, important for targeting the 3CLpro of enteroviruses. As the S2 pocket Mpro of betacoronaviruses, like SARS-CoV and SARS-CoV-2, presents considerable adaptability to smaller inhibitor moieties, the less bulky cyclopropyl group was inserted trying to improve the antiviral activity (**117**, Figure 35). The novel compound demonstrated improved inhibitory activity against the purified recombinant SARS-CoV-2 Mpro (IC_50_ 0.67 ± 0.18 μM), SARS-CoV Mpro (IC_50_ 0.90 ± 0.29 μM), and MERS-CoV Mpro (IC_50_ 0.58 ± 0.22 μM). RNA replication was evaluated in the replicon assay, showing an EC_50_ value of 1.75 ± 0.25 μM. Antiviral activity in human Calu3 cells infected with the novel coronavirus, SARS-CoV-2, was in the low micromolar range (EC_50_ 4–5 μM). The removal of the Boc group at P3 (**118**, Figure 35) consisted of a complete lack of activity, suggesting the importance of a lipophilic moiety at this position in order to pass the cellular membrane.^194^ For what concerns the ADME properties, both **116** and **117** demonstrated a good stability in mouse and human microsomes and good pharmacokinetic profiles. The lung distribution after nebulizer administration at 3 mg/kg in mice was a value of 33 ng/g, showing that direct administration in the most affected tissue is possible and tolerable.^194^

#### 3.2.2 Calpain Inhibitors

Calpains are calcium-activated cysteine proteases widely distributed in animal cells. The two major isoforms are calpain-1 and calpain-2, which require micro- and millimolar concentration of calcium, respectively, for an optimal enzyme activity in vitro. In physiological conditions, calpains are involved in several processes including platelet activation, T-cell activation, T-cell migration, signal transduction pathways, cell differentiation and proliferation, and apoptosis. An enhanced calpain activity resulted in unregulated proteolysis and anomalous activation of signaling cascades, leading to cellular damage and to cell death. Inhibitors of calpain, after pathological insult, produced cell- and organ-protective effects, suggesting the potential role of calpain as a therapeutic target for several degenerative disorders.^195^

A series of dipeptidyl α-ketoamide derivatives of general structure R_1_-l-Leu-D,L-AA-CONHR_2_ has been developed as inhibitors for the cysteine proteases calpain-1, calpain-2, and cathepsin B by Powers et al.^196^ Peptide derivatives containing electrophilic α-ketoamide group were shown to reversibly inhibit cysteine proteases by forming a hemithioacetal with the SH group of the active site cysteine after a nucleophilic addition of the enzyme to the α-ketoamide.^197^ In their previous study, Li et al. described a series of dipeptidyl and tripeptidyl α-ketoamides, showing that *N*-monosubstitution on the α-ketoamide nitrogen yields compounds with a higher inhibitory potency with respect to the corresponding *N*,*N*-disubstituted α-ketoamides, suggesting the presence of an hydrogen bond between the NH and an amino acid residue of the active site of calpain.^197^ Moreover, the higher activity shown by α-ketoamides bearing hydrophobic groups suggested the existence of a hydrophobic pocket in the active site. Starting from these results, with the aim to further explore the H-bonding ability of this class of compounds, a series of α-ketoamides featuring one or several heteroatoms was developed; moreover, molecules incorporating heteroatoms into aromatic structures at R_2_-position were studied to probe the hydrophobic pocket. In order to investigate the H-bond ability and the hydrophobicity of another region of the active site, different heterocyclic or nonheterocyclic aromatic groups were introduced at the R_1_-position, whereas a α-aminobutyric acid (Abu), a phenylalanine (Phe), or a norvaline (Nva) was chosen as AA substituents.^196^ Most of compounds strongly inhibited calpain-2; also, calpain-1 was effectively inhibited by these derivatives, but only a few compounds showed a very low *K*_i_ value. Most of the compounds weakly inhibited cathepsin B. Regarding the amino acids, Nva appeared to be the best choice for calpain-1 and Abu for calpain-2, although in two cases the substitution of Abu with Phe produced an increase in affinity. Finally, changing substituents at the R_2_-position resulted in only a slight variation of activity toward calpain-1; however, in the case of calpain-2, the presence of a hydrophobic pocket in the active site of this enzyme was confirmed by the fact that the α-ketoamides featuring R_2_=CH_2_CH_2_–Ph or R_2_=CHOH–CH_2_Ph were excellent calpain-2 inhibitors.^196^

Several years later, one of these selective calpain-2 inhibitors, (*Z*-Leu-Abu-CONH-CH_2_-C_6_H_3_(3,5-(OMe)_2_), was selected and tested for its effect on the impairment in long-term potentiation (LPT), evidencing the opposite effects exerted by calpain-1 and calpain-2.^198^ While calpain-1 is positively involved in some types of learning and memory, calpain-2 plays a negative role in the same processes. These results demonstrated that a selective calpain-2 inhibitor could represent a useful tool to treat several disorders related to cognition impairment.^198^

**Figure 36:**
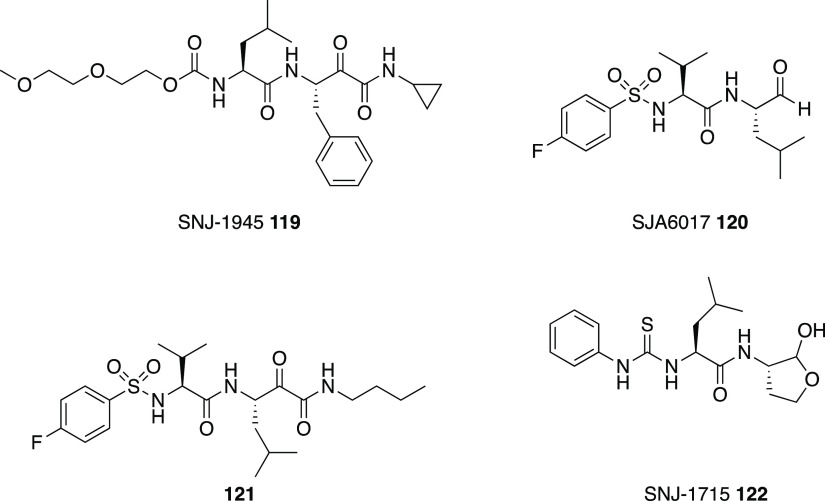
Calpain inhibitors developed by Shirasaki et al.^13^

SNJ-1945 (**119**, Figure 36) emerged from an optimization campaign of the dipeptidyl aldehyde inhibitor SJA6017 (**120**, Figure 36), which showed efficacy as anticataract agent in lens culture models but poor oral bioavailability.^13^ As it seemed that this result could be ascribed to the too-easily metabolized aldehyde moiety, researchers introduced the α-ketoamide obtaining compound **121** with comparable inhibitory activity of **120**, higher cellular permeability, and higher metabolic stability, but very low solubility, which resulted in insufficient oral bioavailability during the pharmacokinetic studies conducted in monkeys. Introduction of cyclopropyl moiety at P1′ and ethylene glycol chain at P3 resulted in **119** (Figure 36).^13^

**Figure 37:**
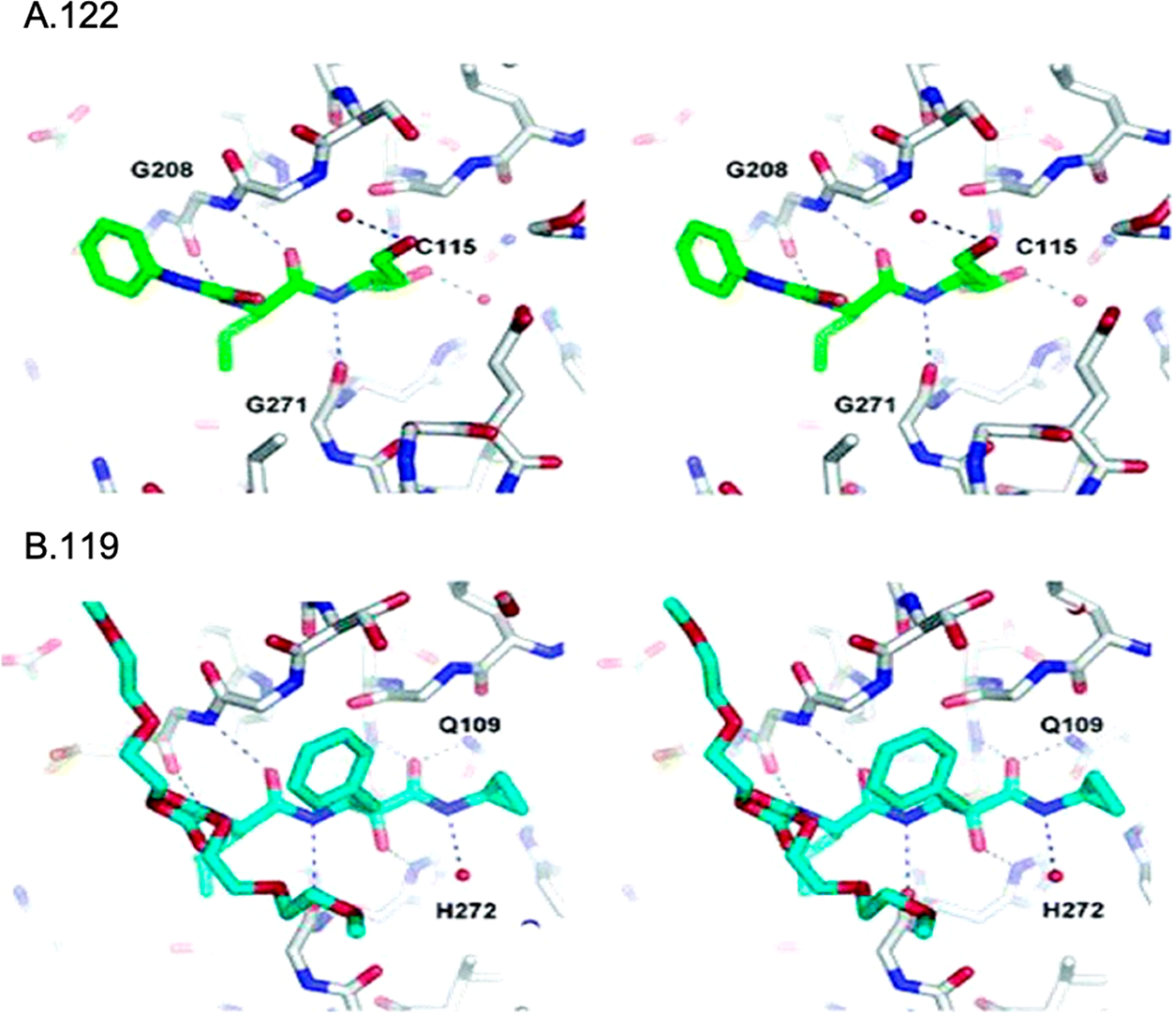
Hydrogen bonding interactions between μI–II and its inhibitors. All intermolecular polar interactions between μI–II and (A) **122** and (B) **119** of <3 Å are shown.^10^ Reproduced from ref (10). Copyright 2006 American Chemical Society.

Comparison of X-ray crystal structures of compound **119** and SNJ-1715 (**122**, Figure 37) bearing a cyclic hemiacetal (a “masked” aldehyde) as an electrophilic warhead, showed the larger number of polar contacts and the stronger hydrogen bonding achieved by the former in the active site.^10^ As reported in Figure 37, the aldehyde warhead of **122** forms a stable hemithioacetal bond with the catalytic cysteine and the resulting hydroxy group directed toward the oxyanion hole formed by Gln109 and Cys115. **119** acts similarly, but the hydroxy group resulted from the nucleophilic attack onto the α-carbonyl forms two hydrogen bonds: one potentially strong with His272 and one presumed weaker with the backbone oxygen of Gly271. The intermediate is further stabilized by two hydrogen bonds between the carbonyl oxygen and the oxyanion hole, Gln109 and Cys115.^10^

**Figure 38:**
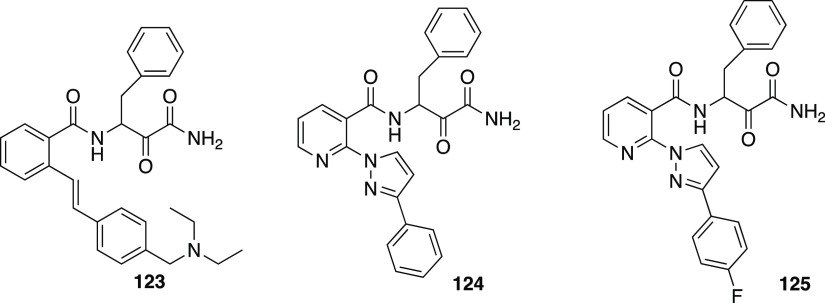
Structures of calpain inhibitors **123**-**125** by Moeller et al.^11,18^

In 2014, Banik et al. showed that calpain is a useful target for the treatment of inflammatory and neurodegenerative events associated with experimental autoimmune encephalomyelitis (EAE) and multiple sclerosis (MS). They demonstrated that orally administration of **119** reduced inflammation by increasing regulatory T cells (Tregs) and by decreasing proinflammatory Th1/Th17 cells, as well as neurodegeneration by reducing the gliosis, axonal damage, and cell death.^199^

Subsequently, the same research group reported that neuroblastoma cells SH-SY5Y, when differentiated into dopaminergic (SH-SY5Y-DA) and cholinergic (SH-SY5Y-ChAT) phenotypes after exposure to mitochondrial toxins MPP^+^ and rotenone, showed calpain activation and highlighted the activation of calpain as a common denominator in various phenotypes in models of Parkinsonism. Moreover, they demonstrated that the calpain inhibitor **119** exerted significant neuroprotection, attenuated the ROS production in dopaminergic phenotype while in cholinergic one down-regulated COX-2, caspase-1, and cleaved caspase-1 p10.^200^

In 2003, Moeller et al. developed a nonpeptidic ketoamide-based calpain inhibitor (**123**, Figure 38).^11^ Although **123** strongly inhibited calpains, it was not selective toward other cysteine proteases (Cal-1 *K*_i_ 56 nM, Cat B *K*_i_ 28 nM, Cat K *K*_i_ 1.8 nM, Cat L *K*_i_ 137 nM, Cat S *K*_i_ 3290 nM; inhibition at 10 mM: Cat C 94%, Cat H 84%), thus preventing further advancement of this compound.^11^

Recently, the same research group reported a library of ketoamide-based 2-(3-phenyl-1*H*-pyrazol-1-yl)nicotinamides as selective calpain inhibitors. The most promising and selective calpain-1 inhibitors (**124**: Cal-1 *K*_i_ 18 nM and **125**: Cal-1 *K*_i_ 34 nM) are presented in Figure 38. These compounds showed high cell permeability, microsomial stability, and functional efficacy in cellular assays.^18^

A subsequent first-in-human Phase I study showed low bioavailability (*F*_e_ ≈ 10%), short effective half-life, and significant formation of the hydroxyamide metabolite (95-fold excess of hydroxyamide metabolite to parent).^18^

The α-ketoamide moiety was then further modified on the nitrogen with a set of different alkyl-, *O*-alkyl-, aryl-, and heteroaryl residues to identify calpain inhibitors with enhanced stability against carbonyl reduction, which should translate into an improved pharmacokinetic profile in humans.^201^

*N*-Alkyl extension presented a strong increase in cytosolic stability but also a significant reduction in calpain inhibition, as in compounds **126**–**130** (Table 12). Aromatic moieties were better tolerated, in terms of calpain inhibition, as exemplified by compounds **129** and **130** (Table 12). On the other hand, these analogues were not suitable for further advancement due to insufficient stability in liver microsomes, probably due to the enhanced lipophilicity. P1′ *N*-alkoxy products (**131**–**133**, Table 12) were generally more potent than the corresponding *N*-alkyl analogues.^201^ From this series containing more than 80 derivatives, the cycloalkyl amide **128** and *N*-methoxy amide **131** emerged as those molecules with the best balance between calpain inhibition, microsomal and cytosolic stability, and selectivity versus cysteine protease cathepsins. Even if they showed a diminished calpain inhibition in vitro, IC_50_ values of 750 (**128**) and 2150 nM (**131**) were comparable to primary amide **124** in terms of cellular efficacy.^201^

**Table 12:**
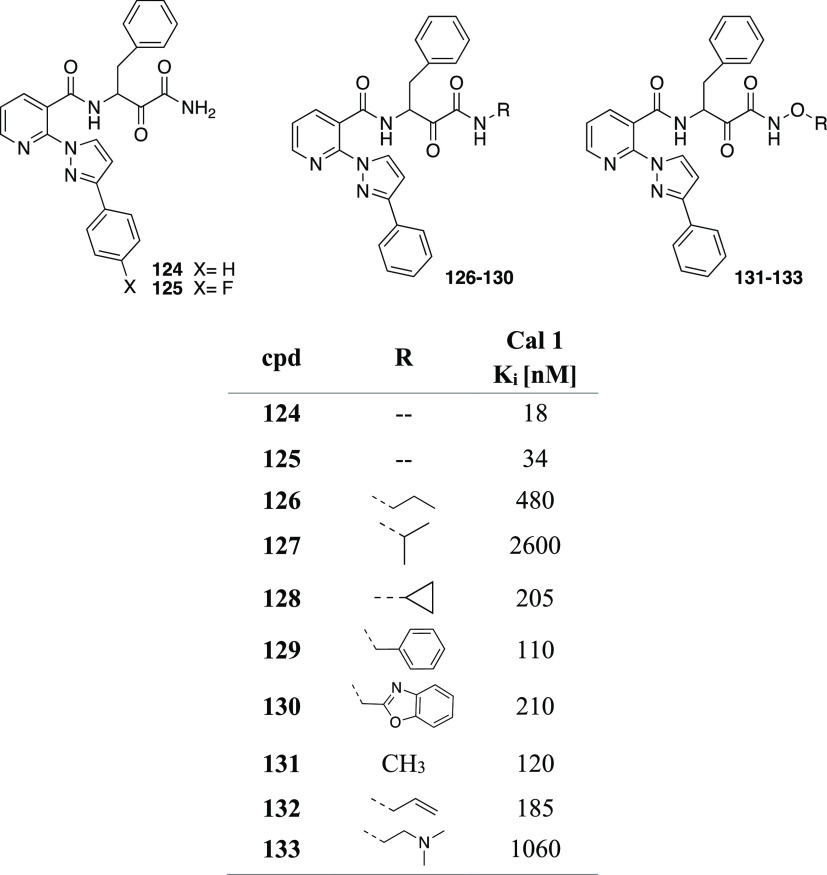
Inhibition of Calpain 1 and Microsomal and Cytosolic Stability of Compounds **124**–**133**^a^

a Data taken from Kling et al.^201^

In another series of compounds, researchers shifted their attention to modifications at portion P1, P2, and P3 of the pharmacophore.^202^ Inspired by previously published research on peptide-based aldehyde inhibitors comprising proline mimetics in P2 position showing improved cathepsin B selectivity,^203,204^ researchers identified compound **134** (Table 13) as lead for further investigations that showed favorable selectivity versus the closely related cathepsins B, K, L, and S. Systematic modifications at P1 did not produce any desired enhancement, so the team investigated the SAR of P3 modifications (**135**–**136**, Table 13). The benzyl moiety at the P3 position of **134** was confirmed to be the best one, so several substitutions on the ring were tried, with 2,6-disubstitution yielding the most potent and selective analogue in this series (**139**, Table 13).^202^

**Table 13:**
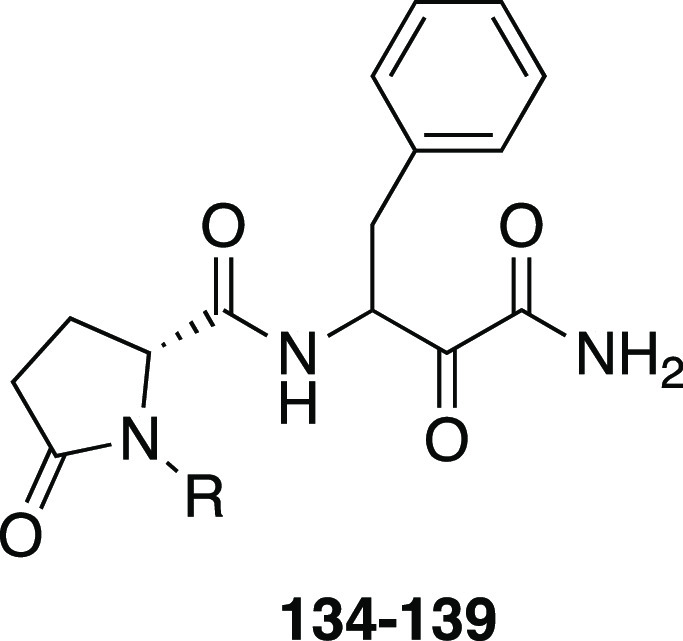

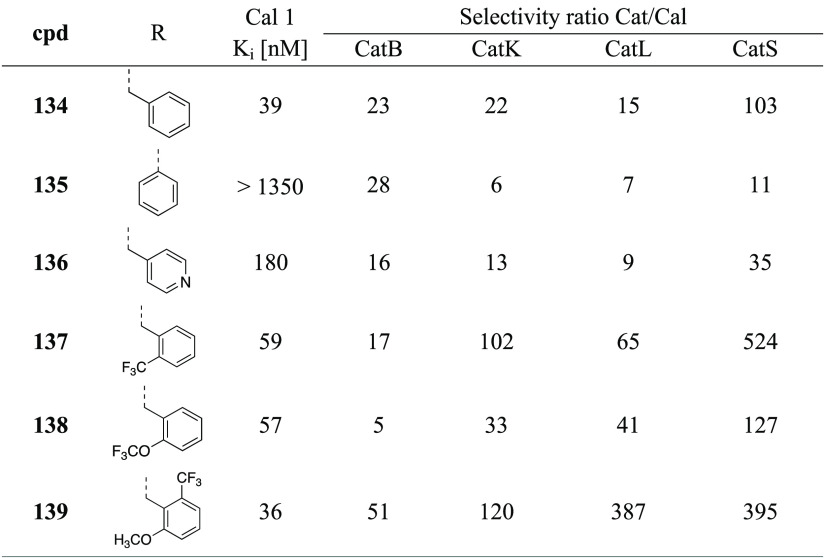
Inhibition of Calpain 1 and Cathepsin Selectivity for Compounds **134**–**139**^a^

a Data taken from Jantos et al.^202^

Several P1′ alkyl, *o*-alkyl, aryl, and heteroaryl amides were then synthesized (as examples, **140**–**143** in Table 14) to increment the cytosolic stability,^201^ even though the ability to inhibit the primary target for most of the analogues decreased. Some aromatic P1′ modifications (**141**–**142**) had a positive impact on cathepsin selectivity while also retaining calpain inhibition, but the advancement was abandoned because of the low stability in liver microsomes.^202^*N*-Cyclopropylamide **140** displayed the best profile balancing potency, selectivity, and metabolic stability and was then further characterized in preclinical models relevant to AD, showing efficacy with respect to prevention of NMDA-induced neurodegeneration and Aβ-induced synaptic dysfunction. Compound **140** advanced in clinical phase I studies as Alicapistat (ABT-957).^197^ However, the study was unable to demonstrate a pharmacodynamic effect in the CNS, posing a major risk in further clinical development of the molecule for AD treatment.^205^

**Table 14:**
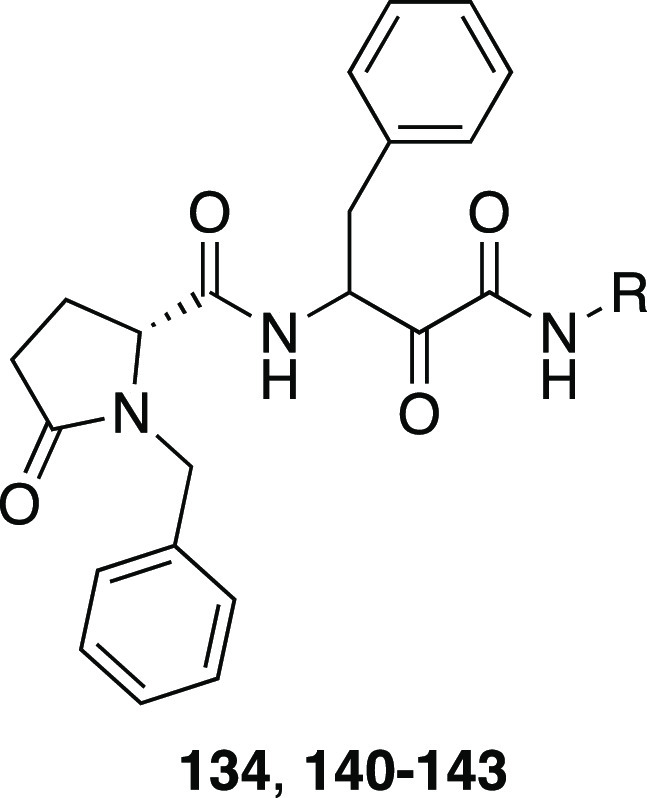

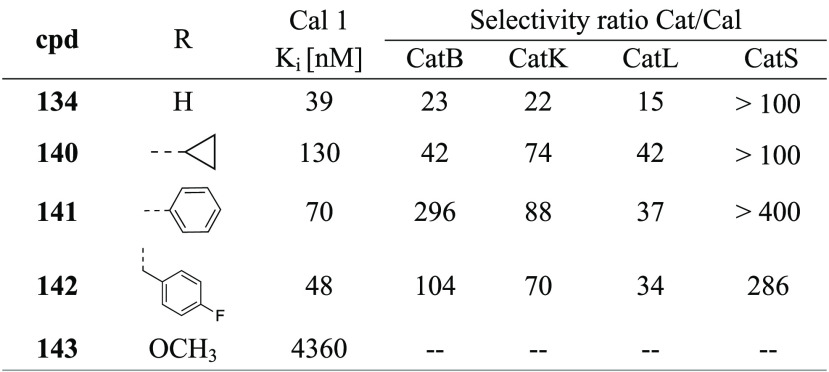
Inhibition of Calpain 1, Cathepsin Selectivity, and in-Vitro Clearance for Compounds **134** and **140**–**143**^a^

a Data are taken from Kling et al.^201^

**Figure 39:**
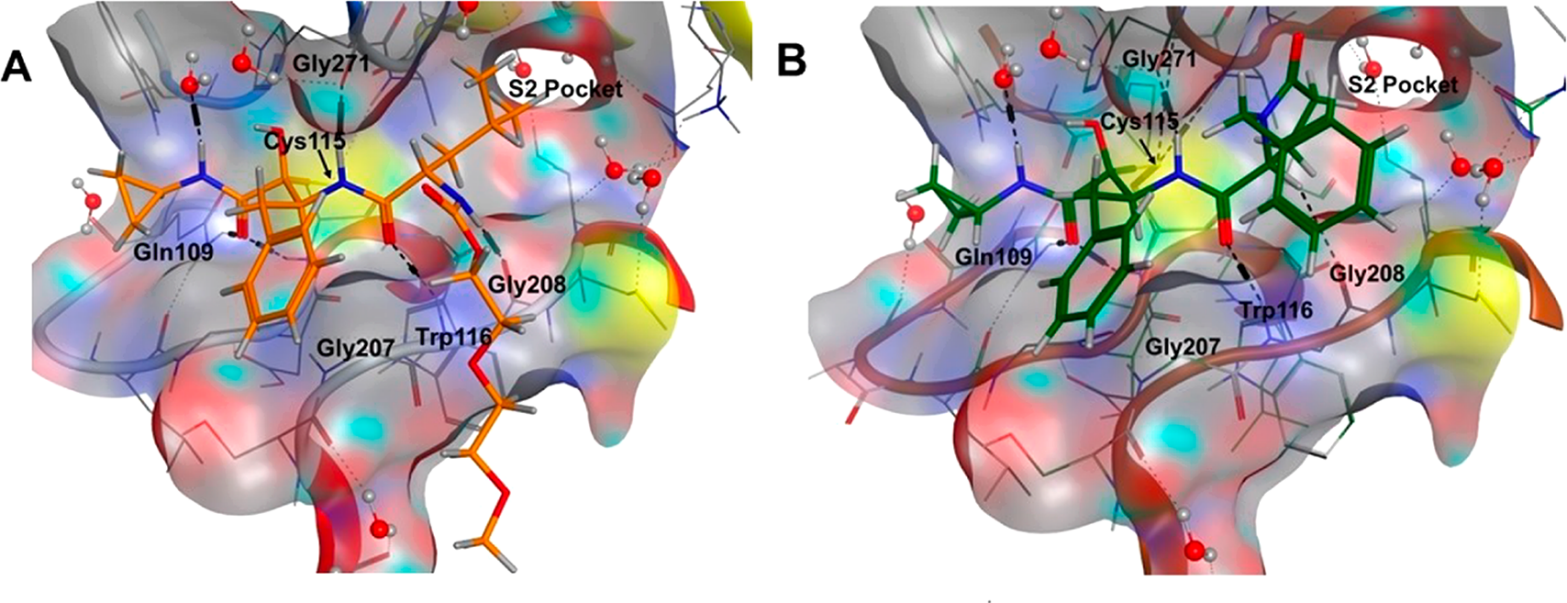
Binding of **119** (A) and **140** (B) in the active site of calpain-1.^206^ Reproduced with permission from ref (206). Copyright 2020 Wiley-VCH GmbH.

Modeling studies using an X-ray crystal structure of calpain-1 with the known α-ketoamide-based inhibitor **119** (SNJ-1945 Figure 37) showed that the binding mode of the enantiomer *R,S* of Alicapistat **140** was similar to that of the original ligand.^206^ As reported in Figure 39, the nucleophilic attack of Cys115 on the α-keto carbonyl of **140** leads to the formation of the tetrahedral adduct, as in compound **119**. The formed oxyanion is subsequently protonated by His272. The adduct is stabilized through hydrogen bond interactions between the carbonyl oxygen of the amide portion and the backbone amides of canonical residues Gln109 and Cys115 and between the hydroxyl group and His272 and Gly271 (Figure 39A,B).^10^ The oxopyrrolidine moiety stays in the S2 pocket similarly to the leucine residue of **119**. Additional hydrogen bonds are formed by both NH-groups and the carbonyl oxygen in the P2 region of **140** with Gly271 and Gly208.^10^

## 4 Future Perspectives and Conclusion

The purpose of this perspective was to highlight to medicinal chemists how the α-ketoamide functional group may represent a valuable option within drug discovery programs to develop compounds with favorable biological activities, low toxicity, and promising PK and drug-like properties, thus helping to face biological targets of increased complexity. Furthermore, this motif is suitable to a great number of different decorations at both the amide nitrogen atom and α-keto group that may influence the molecular geometry, the specificity for a certain target, and the pharmacokinetic properties of the developed derivatives that aim to produce a specific therapeutic effect. These peculiar properties of the α-ketoamide function make it a privileged structure in medicinal chemistry that have led to the development of a wide array of compounds that have shown a variety of pharmacological activities. In recent years, medicinal chemists have elegantly exploited the α-ketoamide to identify molecules with clinical potential, primarily as sedative/hypnotics, anxiolytics, antitumorals, antibacterials, antivirals, and antiprion.

Bioisosterism is a commonly employed approach in the rational modification of lead compounds to increase potency or enhance selectivity, as well as to improve pharmacokinetic properties and/or reduce toxicity and acquire novel chemical space to secure intellectual property. The introduction of a bioisostere in a new molecule may lead to structural changes in molecular size, shape, p*K*_a_, electronic distribution, polarizability, or dipole that can be favorably exploited to ameliorate the biological activity of the parent compound.

In our view, the α-ketoamide moiety may be regarded as a bioisostere of heterocyclic rings of which the medicinal chemist may take advantage to modulate the conformation of lead compounds by decreasing their structural rigidity and conferring the capacity to establish stronger interactions with the target protein. Moreover, the two electron-rich oxygen atoms of the α-ketoamide may represent further points of interaction with the target protein, thus playing a crucial role in enhancing the affinity and selectivity of the compound for the specific protein, especially if the protein is prone to form hydrogen bonds. This strategy has been successfully employed to obtain the BzR ligand IGAs as bioisosteres of β-carbolines.

Still in the vein of bioisosterism, the pseudoplanar α-ketoamide may replace an acetamide moiety conferring a constraint to its structural flexibility that, hopefully, can permit the whole molecule to fit more securely into the receptor protein, as exemplified by the TSPO ligand PIGAs. However, the possibility cannot be ruled out of the α-ketoamide in place of the acetamide to add further points of interaction with the protein, especially by the electron-rich α-keto oxygen atom.

Furthermore, the nonreactive α-ketoamide has been employed to overcome the limitations associated with peptides. These limitations include susceptibility to degradation by proteases or peptidases to obtain small molecular peptidomimics with enhanced metabolic stability, lower in vivo toxicity, and better selective action on biological targets.

This is only one facet of the attractiveness of the α-ketoamide in the medicinal chemistry field. The key to its versatility is undoubtedly that its structural motif possesses two nucleophilic reaction sites and two electrophilic centers that represent potential, and often crucial, interaction points with the target proteins.

Thus, the α-ketoamide may also be exploited by the medicinal chemist as a reactive moiety in potential drugs: it is sterically more adaptable than other warheads like Michael acceptors and aldehydes, and possesses better pharmacokinetic properties, such as improved membrane permeability and enhanced stability toward plasma esterases, than α-ketoesters.^11,12^ It also demonstrates higher resistance against proteolytic cleavage^5^ and superior chemical and metabolic stability than the aldehyde derivatives, due to less reactivity.^12−14^ The ability of the 2-oxoamide moiety to resemble both a scissile amide and ester bond makes it suitable to be included as an electrophilic warhead in designing inhibitors that are analogues of substrates for those enzymes responsible for catalyzing the cleavage of those type of chemical bonds through a nucleophilic attack. Particularly, serine and cysteine proteases have proved over the years to be suitable targets in terms of rational design of novel inhibitors featuring the α-ketoamide moiety. An example for all is represented by the development of reversible cysteine protease (calpain) inhibitors: the carbonyl reactive group of the α-ketoamide was able to form a hemithioacetal with the SH group of the active site cysteine by nucleophilic addition.

Finally, it should be noted that several α-ketoamide-based libraries with interesting biological properties are reported in the literature, which are developed starting from a lead compound identified by a virtual screening campaign. Although, in these cases, a rationale for exploiting the α-ketoamide moiety cannot be detected, ex-post SAR studies revealed the crucial role played by this group in the interaction with the target protein.

In conclusion, the objective of the present work is to emphasize that the α-ketoamide is a quite unique moiety, that is, a privileged structure, as it may be involved in critical drug–target interactions and modulation of drug properties. We highlighted its peculiar role in medicinal chemistry, reviewing its physicochemical properties and describing its involvement in the formation of donor–acceptor hydrogen bonding interactions and reactivity with the target receptors or enzymes.

Finally, this report provides exciting perspectives on existing data and that exploiting the α-ketoamide moiety in modern medicinal chemistry will help to open new avenues in drug design and development, resulting in more efficient drug candidates introduced onto the market and into the clinical pipeline.

## Abbreviations Used

3Cpro: protease 3C
3CLpro: 3C-like protease
AdPLA: adipose-PLA2
AHL: *N*-acyl homoserine lactone
AKR: aldo-keto reductase
AMP: antimicrobial peptide
ARDS: adult respiratory distress syndrome
Bz: benzodiazepine
BzR: benzodiazepine receptor
Cal: calpain
Cat: cathepsin
CBR: central benzodiazepine receptor
CC50: 50% cytotoxic concentration
CCR5: C–C chemokine receptor type 5
CD4: cluster of differentiation 4
cPLA2: cytosolic Ca^2+^-dependent PLA2
cpd: compound
CREB: cAMP-response element binding protein
CXCR4: C-X-C motif chemokine receptor 4
CSC: cancer stem cell
DXMS: deuterium exchange mass spectrometry
FRET: fluorescence resonance energy transfer
Δψm: mitochondrial membrane potential
FDA: USA Food and Drug Administration
GABAA: γ-aminobutyric acid
GBM: glioblastoma multiforme
GRK2: G protein-coupled receptor kinase 2
HAART: highly active antiretroviral therapy
HGL: human gastric lipase
HIV: human immunodeficiency virus
HLM: human liver microsomes
iPLA2: cytosolic Ca^2+^-independent PLA2
LPLA2: lysosomal PLA2
MD: molecular dynamic
MDM2: murine double minute 2
MDR: medium-chain dehydrogenase/reductase
MIC: minimum inhibitory activity
Mpro: main protease
MPTP: mitochondrial permeability transition pore
MSC: mesenchymal stem cell
PAF-AH: platelet activating factor acetyl hydrolase
PEG-IFNα-2a: pegylated interferon alfa-2a
PL: pancreatic lipase
PK: pharmacokinetic
PLA2: phospholipase A2
PPL: porcine pancreatic lipase
PrPC: normal cellular prion protein
PrPSc: scrapie prion protein
QR: quinone reductase
QS: quorum sensing
QSI: quorum sensing inhibitor
RT: residence time
SAR: structure–activity relationship
SDR: short-chain dehydrogenase/reductase
SMAMP mimic: small molecular antimicrobial peptidomimic
sPLA2: secreted PLA2
SEM: standard error of the mean
TSEs: transmissible spongiform encephalopathies
TSPO: translocator protein
